# Transcriptomic Analysis of Differentially Expressed Genes during Flower Organ Development in Genetic Male Sterile and Male Fertile *Tagetes erecta* by Digital Gene-Expression Profiling

**DOI:** 10.1371/journal.pone.0150892

**Published:** 2016-03-03

**Authors:** Ye Ai, Qinghua Zhang, Weining Wang, Chunling Zhang, Zhe Cao, Manzhu Bao, Yanhong He

**Affiliations:** 1 Key Laboratory of Horticultural Plant Biology, Ministry of Education, College of Horticulture and Forestry Sciences, Huazhong Agricultural University, Wuhan 430070, Hubei, China; 2 College of Landscape Architecture, Fujian Agriculture and Forestry University, 15# Shangxiadian Road, Cangshan District, Fuzhou 350002, Fujian, China; 3 College of Forestry, Fujian Agriculture and Forestry University, 15# Shangxiadian Road, Cangshan District, Fuzhou 350002, Fujian, China; 4 Gulf Coast Research and Education Center, Institute of Food and Agricultural Sciences, University of Florida, Wimauma, Florida 33598, United States of America; University of Naples Federico II, ITALY

## Abstract

*Tagetes erecta* is an important commercial plant of Asteraceae family. The male sterile (MS) and male fertile (MF) two-type lines of *T*. *erecta* have been utilized in F_1_ hybrid production for many years, but no report has been made to identify the genes that specify its male sterility that is caused by homeotic conversion of floral organs. In this study, transcriptome assembly and digital gene expression profiling were performed to generate expression profiles of MS and MF plants. A cDNA library was generated from an equal mixture of RNA isolated from MS and MF flower buds (1 mm and 4 mm in diameter). Totally, 87,473,431 clean tags were obtained and assembled into 128,937 transcripts among which 65,857 unigenes were identified with an average length of 1,188 bp. About 52% of unigenes (34,176) were annotated in Nr, Nt, Pfam, KOG/COG, Swiss-Prot, KO (KEGG Ortholog database) and/or GO. Taking the above transcriptome as reference, 125 differentially expressed genes were detected in both developmental stages of MS and MF flower buds. MADS-box genes were presumed to be highly related to male sterility in *T*. *erecta* based on histological and cytological observations. Twelve MADS-box genes showed significantly different expression levels in flower buds 4 mm in diameter, whereas only one gene expressed significantly different in flower buds 1 mm in diameter between MS and MF plants. This is the first transcriptome analysis in *T*. *erecta* and will provide a valuable resource for future genomic studies, especially in flower organ development and/or differentiation.

## Introduction

Plants with male sterility have been applied effectively and economically in plant breeding for pollination control, especially in Asteraceae family, which has the unique structure of terminal capitulum that contains hundreds of florets of two different types, ray florets in the periphery and disk florets in the center. Breeders are looking for the male sterile (MS) plants with defective anthers, and degenerated petals of ray and disc florets to save the expense on manual emasculation [1, 2]. *Tagetes erecta*, a member of the Asteraceae family, is an important commercial plant used for ornamental, industrial and medicinal purposes [3–5]. Fortunately, MS line of *T*. *erecta* was found in nature, in which the petals of florets developed into filament-like structures and the stamens became yellow filaments with no pollen formed [6]. The degeneration of petals and stamens seems to be a perfect trait for pollination control and the MS lines of *T*. *erecta* have been utilized successfully in F_1_ hybrid production [7, 8].

The associated phenotypic manifestations of male sterility include the absence or abnormality of male organs, failure to form normal sporogenous tissues, pollen abortion, failure of stamen dehiscence, and the inability of mature pollen to germinate on compatible stigma [9, 10]. The previous histological and cytological analysis found that, in *T*. *erecta*, the petals of the ray and disc florets of the MS plant developed into sepal-like, while the stamens were partially converted to styles [11]. It indicated that the male sterility in *T*. *erecta* is probably caused by the homeotic conversion of stamens into other floral organ structures, i.e. corresponding to the category of male organ abnormality. Based on the ABCDE model of floral organ development, the homeotic conversion of floral organs is due to the mutation of MADS-box A-, B-, C-, D- and E-class genes [12]. The homeotic conversion in *T*. *erecta* might be, at least in part, the result of mutation of MADS-box genes [11]. However, this suggestion needs to be further investigated and validated. And more studies are needed to elucidate the molecular mechanism of male sterility in *T*. *erecta*.

Next generation sequencing techniques had improved the efficiency and reduced the cost of sequencing, hence accelerated gene expression profile comparison and gene discovery [13]. Transcriptome assembly is a valuable tool to study transcriptomics, in which the expressed genes can almost cover the entire transcriptome when assembled together [14, 15]. Digital gene expression (DGE) analysis, on the other hand, is a powerful tool to identify and quantify gene expression on the whole genome level, in which differentially expressed genes and their related pathways can be analyzed comprehensively [16–19]. Combining transcriptome assembly and DGE approaches has facilitated the identification of candidate genes in non-model plants, as it takes the advantages of both, not only enabling large scale gene functional assignment via large sequenced transcriptome library assembly, but making it possible to easily perform quantitative gene expression comparisons without potential biases, thus allowing for a more sensitive and accurate profiling of the transcriptome that more closely resembles cell activity [20–22].

There were many reports about the use of transcriptome assembly and DGE techniques to study the mechanism of male sterility. In sterile *Cybrid Pummelo* (Rutaceae family), a large number of differentially expressed genes were identified at both petal primordia and stamen primordia stages [23]. In *Capsicum annuum* (Solanaceae family), a set of potential candidate genes were found to associate with the formation or abortion of pollen between a cytoplasmic MS line and its near-isogenic restorer line [24]. In sterile *Brassica napus* (Brassicaceae family), many genes were identified to be involved in pollen tube development and growth, pollen wall assembly and modification, pollen exine formation and pollination [25]. In *Gossypium hirsutum* (Malvaceae family), thousands of genes were differentially expressed at the meiosis, tetrad, and uninucleate microspore stages of anthers [26, 27]. These findings provided a better understanding of the regulatory network involved in stamen, anther and pollen development. To our knowledge, in Asteraceae family, there has been no transcriptomic analysis of differentially expressed genes related to spontaneous male sterility caused by homeotic conversion.

To generate more complete observations of transcriptome content and find out candidate genes associated with male sterility in *T*. *erecta*, we constructed a reference transcriptome for flower buds of *T*. *erecta* using Illumina Sequencing. Further, we used DGE analysis to compare the gene expression level between the MS and male fertile (MF) flower buds when they grew to 1 mm and 4 mm in diameter. This is the first genome-wide gene expression profiling of male sterility in *T*. *erecta*. The data will provide an invaluable resource for identifying genes involved in flower development and provide insights into the molecular mechanisms of male sterility in *T*. *erecta*.

## Materials and Methods

### Materials

**Fig 1:**
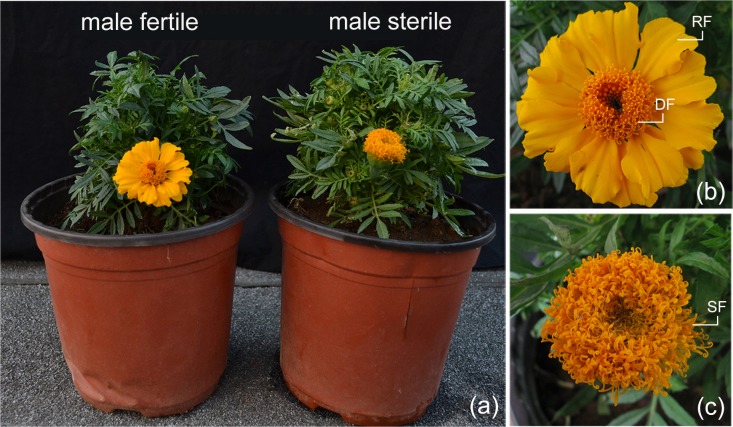
. Morphological characteristics of the male sterile and male fertile two-type line M525AB of *T*. *erecta*. (a) Plant morphology of male sterile plant M525A (right) and male fertile plant M525B (left); (b) Inflorescence morphology of male fertile plant M525B; (c) Inflorescence morphology of male sterile plant M525A. RF: ray floret, DF: disc floret, SF: sterile floret.

The genic MS and MF two-type line M525AB of *T*. *erecta*, derived from an individual natural mutant found in 2004, was maintained by sib-mating [11]. The MS plant named as M525A displayed degenerated petals and stamens (Fig 1A and 1C), while the MF plant labelled as M525B exhibited normal floral organs (Fig 1A and 1B). An F_1_ segregation population was obtained by self-pollination of a single plant M525B in 2013. When two pairs of true leaves emerged, the homozygous MS and homozygous MF plants were identified by the SCAR maker SC4 [11]. Plants were grown in the experimental field of Huazhong Agricultural University (located at 30°28'36.5" North latitude and 114°21'59.4" East longitude), Wuhan, Hubei Province, China.

### Morphology observation

When the plants came into bloom, floret organs and different sizes (0.5 mm, 1 mm, 2 mm, 3 mm, 4 mm, 5 mm, 6 mm, 7 mm and 8 mm) of flower buds from the homozygous MS and homozygous MF plants were collected for morphology observation under a microscope (BX61, Olympus). The floret organs were also examined under a JEOL (JSM-6390LV) scanning electron microscope (SEM) in the Electron Microscopy Laboratory of Huazhong Agricultural University. The operation steps of SEM have been described in detail by Ai et al [28].

### RNA isolation

Based on the morphological analysis, flower buds (1 mm and 4 mm in diameter) were collected from ten homozygous MS plants and ten homozygous MF plants, respectively. Collected buds were frozen immediately in liquid nitrogen and stored at −80°C for RNA extraction. Flower buds were sampled three times (representing three replications) with an interval of ten days in May 2014. Total RNA from each sample was isolated by the Trizol Reagent (Invitrogen), and RNA quality and quantity were determined by a Nano Photometer spectrophotometer (IMPLEN, CA, USA), a Qubit RNA Assay Kit in a Qubit 2.0 Flurometer (Life Technologies, CA, USA) and a Nano 6000 Assay Kit of the Agilent Bioanalyzer 2100 system (Agilent Technologies, CA, USA). A total of 12 μg RNA, 1 μg from each sample, was used as input for transcriptome library construction and 3 μg RNA per sample was used to construct the DGE library.

### Library preparation and sequencing

RNA Samples were sent to Novogene Bioinformatics Technology Co. Ltd (Beijing), where the libraries were constructed and sequenced using Illumina HiSeq 2000 platform. Sequencing libraries were generated using NEBNext Ultra™ RNA Library Prep Kit for Illumina (NEB, USA) following manufacturer’s protocols and index codes were added to attribute sequences to each sample. Short fragments ranging from 270 bp to 340 bp in length were selected by gel purification and amplified through PCR to create the final sequencing library. Then transcriptome sequencing was carried out on an Illumina HiSeq 2000 platform that generated 100 bp paired-end raw reads, while DGE sequencing generated 100 bp single-end raw reads.

### Transcriptome assembly and gene functional annotation

Raw data (raw reads) of fastq format were firstly processed through in-house perl scripts where clean data (clean reads) were obtained by filtering out reads containing adapter, reads with unknown base ‘N’ (where the ‘N’ ratio was more than 10%), and other low quality reads (where the quality score was lower than 5) from raw data. Meanwhile, Q20 and Q30 (proportion of nucleotides with quality value larger than 20 and 30), and GC-content (proportion of guanine and cytosine nucleotides among total nucleotides) were calculated. All the downstream analyses were based on clean data of high quality. Transcriptome assembly was accomplished by Trinity (Release 2012-10-05) with min_kmer_cov set to 2 by default and all other parameters set to default [29].

The longest transcript of each gene was selected as an unigene, and the function of all assembled unigenes was annotated based on the following databases: Nr (NCBI non-redundant protein sequences), Nt (NCBI non-redundant nucleotide sequences), Pfam (Protein family), KOG (euKaryotic Ortholog Groups), Swiss-Prot (A manually annotated and reviewed protein sequence database), KO (KEGG Ortholog database), and GO (Gene Ontology).The unigenes were annotated in public NR, NT, Swiss-Prot and KOG databases using NCBI blast 2.2.28+ [30], and the Nr, Nt and Swiss-Prot databases had a cut-off E-value of 10^−5^, while KOG database had a cut-off E-value of 10^−3^.

### Analysis of DGE tags and bioinformatics

The clean data of DGE were mapped back onto the assembled transcriptome and read count of each gene was obtained from the mapping results by RSEM-1.2.0 [31] for each sample. The bowtie parameter was set at mismatch 2. All read counts were normalized to FPKM (expected number of fragments per kilobase of transcript per million mapped reads) value, representing gene expression level [32]. To examine the reliability of data between replications, the Pearson’s correlation analysis of gene expression among these samples were carried out by the SPSS software.

Differential expression analysis of the samples with three biological replications (two replications for S1) was performed using the DESeq 1.10.1 via the negative binomial distribution. The input values were based on the read counts. The obtained P values were adjusted using the Benjamini and Hochberg’s approach to control false discovery rate [33]. Genes with an adjusted P value < 0.05 calculated by DESeq were regarded differentially expressed [34].

### GO and KEGG pathway enrichment analysis

GO term enrichment analysis of differentially expressed genes (DEGs) was performed using GOseq 1.10.0 based on the wallenius non-central hyper-geometric distribution which could adjust for gene length bias in DEGs [35]. The GO term with P value < 0.05 was defined as significantly enriched GO term. KEGG (Kyoto encyclopedia of genes and genomes) pathway enrichment analysis was performed based on a FDR cut-off value of 0.05 using KOBAS (version 2.0) after the unigenes were mapped to KEGG pathways [36].

### Quantitative real-time PCR analysis

Quantitative real-time PCR (qRT-PCR) analysis was used to verify the expression levels of genes identified in DGE sequencing. The RNA samples used for qRT-PCR assays were the same as used for the DGE experiments. Reverse cDNA for each sample was generated via the PrimeScript RT reagent Kit with gDNA Eraser (TaKaRa Biotechnology, Dalian, China). Real-time PCR was performed with specific primers that were designed based on the selected unigene sequences with Primer 5.0 software. Housekeeping gene β-actin was used as the control gene. All primers are listed in S1 Table. The qRT-PCR was carried out using a SYBR Primix Ex Taq kit (TaKaRa, Dalian, China) following manufacturer’s instructions and was analyzed in the ABI 7500 Real-Time System (Applied Biosystems, USA). The gene expression levels were calculated by ABI Prism 7500 Sequence Detection System Software (Applied Biosystems, USA). Each reaction contained 2 μl cDNA template, 10 μl 2 × SYBR Green Master Mix, 0.4 μl RT reaction mixture, 0.8 μl forward and 0.8 μl reverse primer (10 μmol/μl) and water to a final volume of 20 μl. The PCR amplification was carried out in a 96-well plate with the following cycling parameters: heating for 2 min at 95°C, 40 cycles of denaturation at 95°C for 10 s, annealing for 20 s at 60°C, and extension at 72°C for 35 s. Real-time quantitative PCR was performed in four replications for each sample and data were shown as mean values ± SD (n = 4). Analysis of the relative gene expression data was conducted using the 2^−ΔΔCt^ method.

## Result

### Morphological analysis

**Fig 2:**
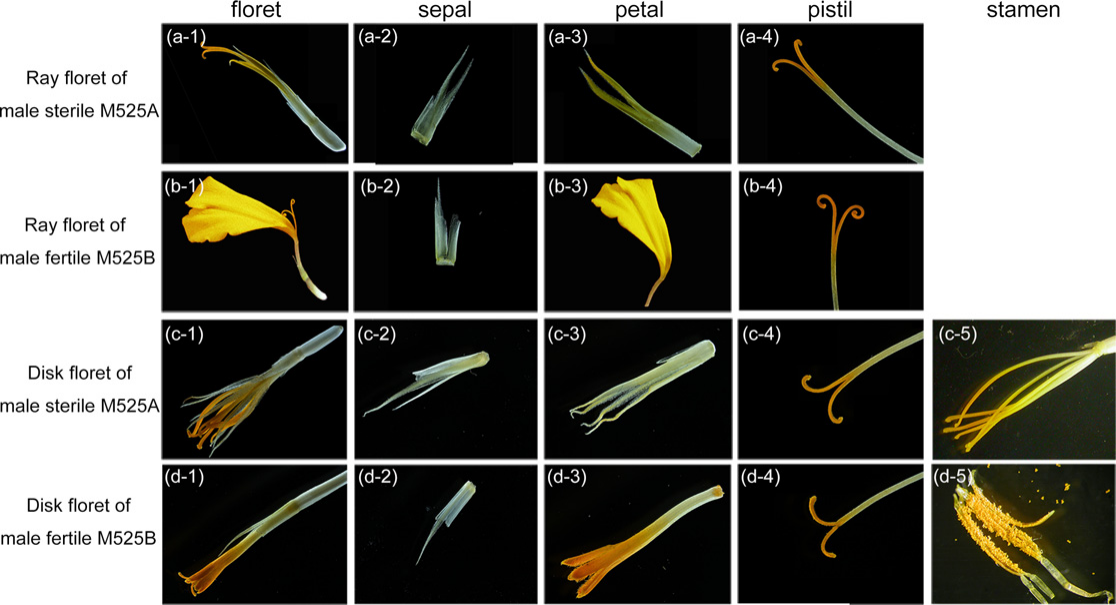
Floret morphology of the male sterile and male fertile two-type line M525AB of *T*. *erecta*. The ray florets of male sterile M525A (a-1) and male fertile M525B (b-1) had three whorls of floral organs, sepal (a-2, b-2), petal (a-3, b-3) and pistil (a-4, b-4), while the petal of ray floret in M525A developed into sepal-like structure. The disk florets of male sterile M525A (c-1) and male fertile M525B (d-1) had four whorls of floral organs, sepal (c-2, d-2), petal (c-3, d-3), stamen (c-5, d-5) and pistil (c-4, d-5). The petals of disc florets in M525A developed into sepal-like structures, while the stamens developed into yellow filaments.

**Fig 3:**
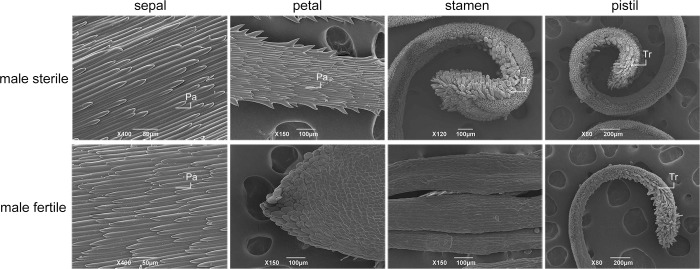
Scanning electron microscope observation of floret morphology of the male sterile and male fertile two-type line M525AB of *T*. *erecta*. The deformed petal of male sterile plant was covered by unusual pappus hairs which were typically found in sepal. The distorted stamen of male sterile plant was covered by trichomes that were only found in stigma walls. Pa: pappus hairs, Tr: trichomes.

*T*. *erecta* has a typical terminal capitulum consisting ray florets in the periphery and disk florets in the center (Fig 1). The ray florets have three whorl floral organs (sepal, petal and pistil), while the disk florets have four whorl floral organs (sepal, petal, stamen and pistil) (Fig 2). Based on the observation of the flower organs, we found that the petals of the ray and disc florets of MS plant developed into sepal-like structures, while the stamens developed into yellow filaments with no pollen formed (Fig 2). Scanning electron microscopy revealed that the deformed petal of MS plant was covered by unusual pappus hairs which were typically found in sepal, not in petal, and the distorted stamen was covered by trichomes that were only seen in stigma walls (Fig 3). From the observation of transverse semi-thin sections [11], we found that the development of the stamen primordia in MS plants failed to differentiate into archesporial cells, sporogenous cells, microspore mother cells, microspore tetrads and pollen grains and the stamens were partially converted to style-like structures. Thus, it is confirmed that male sterility in *T*. *erecta* was due to the inability to form normal archesporial cells and homeotic conversion of floral organs had occurred when the MS floret organs began to differentiate.

**Fig 4:**
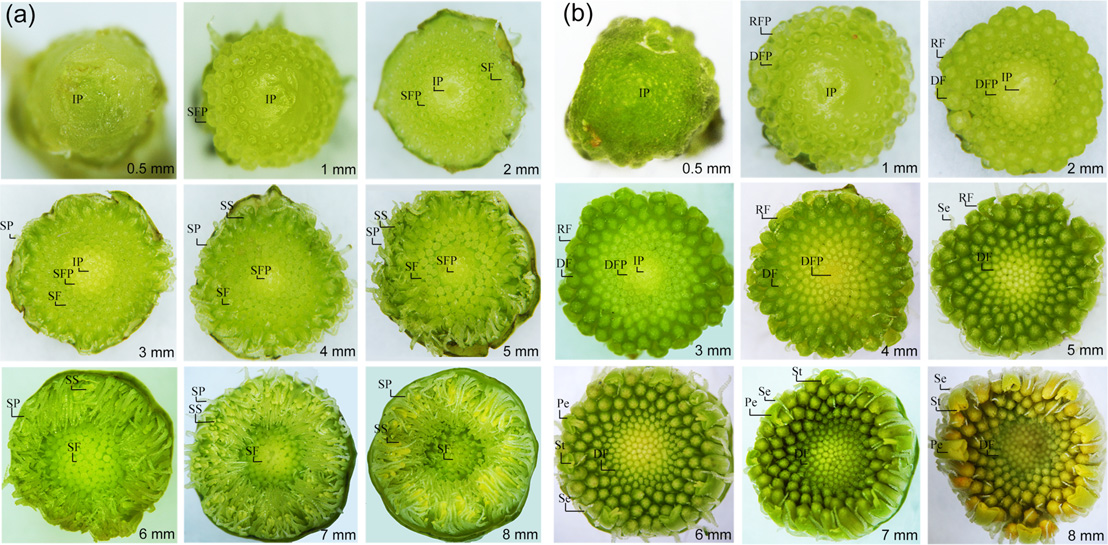
Flower buds development processes of male sterile and male fertile two-type line M525AB of *T*. *erecta*. (a) Developmental process of male sterile M525A’s flower buds from 0.5 mm to 8 mm in diameter; (b) Developmental process of male fertile M525B’s flower buds from 0.5 mm to 8 mm in diameter. IP: inflorescence primordium, SFP: sterile floret primordium, SF: sterile floret, SP: sepal and sepal-like petal of male sterile floret, SS: style-like stamen and stigma of male sterile floret, RFP: ray floret primordium, DFP: disc floret primordium, RF: ray floret, DF: disc floret, Se: sepal of fertile floret, Pe: petal of ray floret, St: stigma of ray floret.

We also observed the developmental process of the flower bud under a stereo microscope. The results showed that only 3–5 rounds of florets in the peripheral of the inflorescence were in the stage of differentiation when the flower bud grew to 1 mm in diameter. When the flower bud grew to 4mm in diameter, the florets in the center began to differentiate and the florets in the peripheral had completed differentiation with their height reaching 3.81±0.03 mm at the outermost (Fig 4). He et al [11] reported that the floret organs completed differentiation process when the height of the floret reached about 4 mm. The homeotic conversion of floral organs took place when the MS floret organs began to differentiate. Based on our observation and former reports, we focused on the differentiation process of floret organs between MS and MF plant, and therefore chose flower buds 1 mm and 4 mm in diameter for transcriptome and digital gene expression (DGE) analysis.

### Generating a reference transcriptome of flower development by Illumina sequencing

To generate a reference transcriptome, RNA was extracted from flower buds (1 mm and 4 mm in diameter) from ten homozygous MS plants and ten homozygous MF plants, and then pooled together for Illumina sequencing. A total of 90,547,072 raw tags were sequenced in the library of *T*. *erecta*. After filtering out reads containing adapter, poly-N and other low quality reads from raw data, 87,473,431 clean tags remained in the library. The base average error rate was 0.03%, and the average Q20 and Q30 values were 97.08% and 90.85%, respectively. In addition, the average GC content was 41.82%. These data showed that the Illumina sequencing was of high quality. There were 128,937 transcripts of clean data assembled using Trinity software [23], and all further analyses were based on these transcripts. The average length of transcript was 1,188 bp, ranging from 201 bp to 13,680 bp. N50 and N90 (Put the splicing transcripts in the order of length. Those cumulative lengths more than 50% or 90% of the length of total splicing transcript are called N50 or N90) were 1,928 bp and 523 bp, respectively. There were 24,158 transcripts longer than 2 kbp (S2 Table). From these transcripts, 65,857 unigenes were identified with an average length of 777 bp, with the longest unigene 13,680 bp, and the shortest 201 bp (N50 was 1,379 bp, and N90 was 296 bp). A total of 5,734 unigenes were longer than 2 kbp (S2 Table).

Based on the annotation results shown in Table 1, there were 28,216 unigenes (42.84%) annotated in NR, 14,893 unigenes (22.61%) annotated in Nt, 8,714 unigenes (13.23%) annotated in KO, 21,085 unigenes (32.01%) annotated in SwissProt; 20,711 unigenes (31.44%) annotated in PFAM; 23,079 unigenes (35.04%) annotated in GO; 10,646 unigenes (16.16%) annotated in KOG. In summary, there were 3,481 unigenes (5.28%) annotated in all databases, and 34,176 unigenes (51.89%) annotated in at least one database.

**Table 1:** Number of unigenes annotated in databases.

Databases	Number of Unigenes	Percentage (%)
Annotated in Nr	28,216	42.84
Annotated in Nt	14,893	22.61
Annotated in KO	8,714	13.23
Annotated in SwissProt	21,085	32.01
Annotated in Pfam	20,711	31.44
Annotated in GO	23,079	35.04
Annotated in KOG	10,646	16.16
Annotated in all Databases	3,481	5.28
Annotated in at least one Database	34,176	51.89
Total Unigenes	65,857	100.00

**Fig 5:**
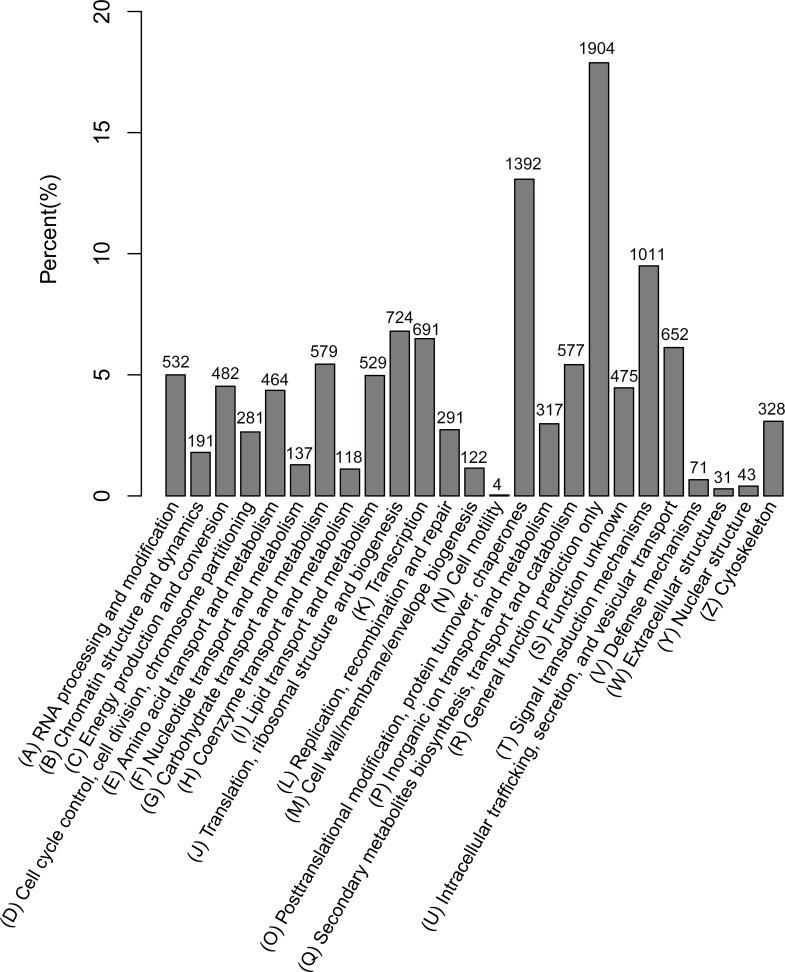
Functional classifications of the assembled unigenes according to the euKaryotic Ortholog Group categories. The x-axis indicated 26 groups of KOG. The y-axis indicated the percentage of the number of annotated genes under a group to the total number of annotated genes.

A total of 10,646 unigenes were annotated in KOG, and these unigenes were categorized into 26 groups of KOG function clusters, among which the ‘general function prediction only’ cluster had the highest number of unigenes (1,904, 15.94%), and the ‘Posttranslational modification, protein turnover, chaperones’ cluster had the second largest number of unigenes (1,392, 11.65%), followed by the ‘signal transduction mechanisms’ cluster (1,011, 8.46%). By contrast, only four unigenes were classified into ‘cell motility’ (Fig 5).

**Fig 6:**
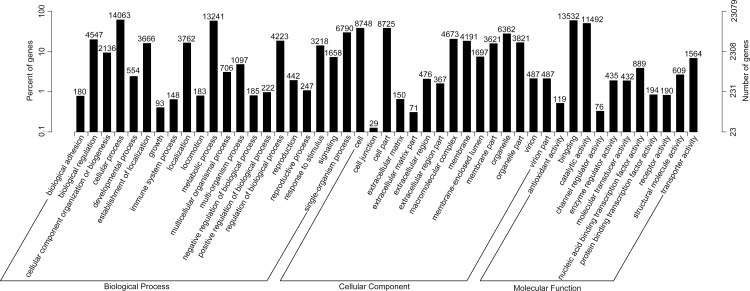
Gene Ontology classifications of the assembled unigenes. The results were categorized into three major categories: cellular component, molecular function, and biological process. The right y-axis indicated the number of genes in a category. The left y-axis indicated the percentage of a specific category of genes in that main category.

Gene Ontology (GO) is an international standardized gene functional classification system that describes properties of genes and their products in any organism. A total of 23,079 unigenes annotated in GO could be categorized into three major categories (cellular component, molecular function and biological process) and 55 subcategories. In the biological process category, the ‘cellular process’ (14,063 unigenes) and the ‘metabolic process’ (13,241 unigenes) were the dominant subcategories. In respect of molecular functions, the major subcategories were ‘binding’ (13,532 unigenes) and ‘catalytic activity’ (11,492 unigenes). In the cellular component category, the ‘cell’ (8,748 unigenes) and “cell part” (8,725 unigenes) were the largest subcategories (Fig 6).

**Fig 7:**
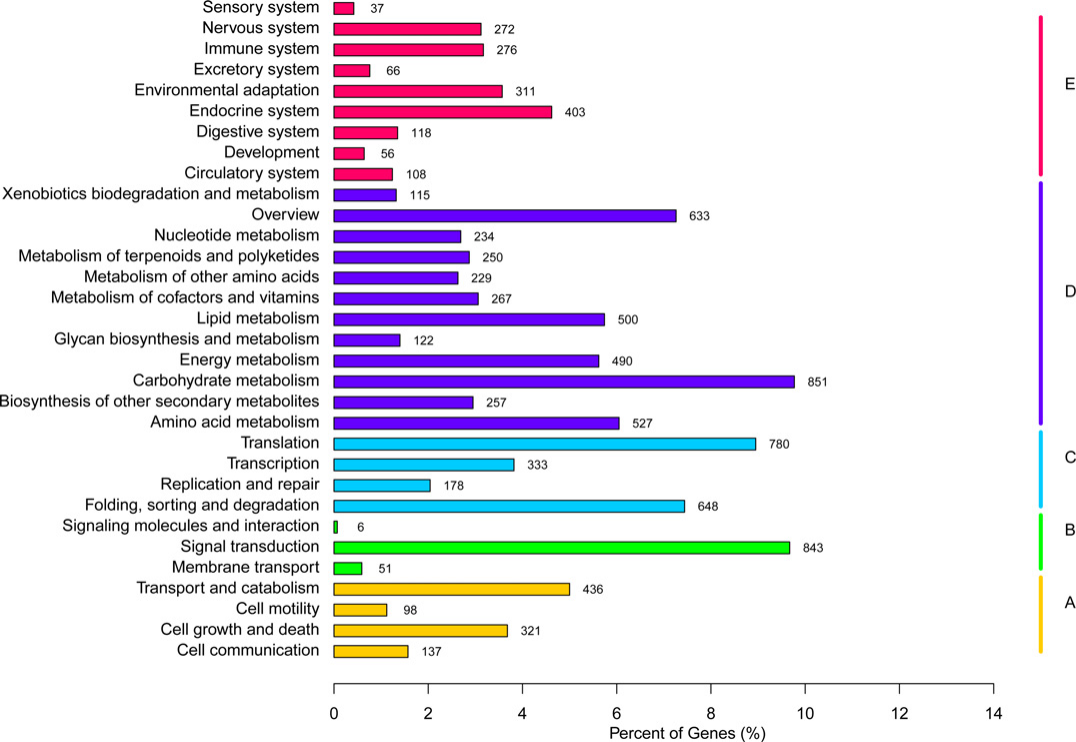
Functional classification of KEGG pathway of assembled unigenes. The KEGG pathways were summarized in five main categories: A, Cellular Processes; B, Environmental Information Processing; C, Genetic Information Processing; D, Metabolism; E, Organismal Systems. The y-axis indicated the name of the KEGG metabolic pathways. The x-axis indicated the percentage of the number of genes annotated under that pathway in the total number of annotated genes.

KEGG pathway has been used to describe the cellular biological molecules that are involved in the metabolic pathways of network diagram, including metabolism pathways, genetic information processing pathways, environmental information processing pathways, cellular process pathways, organismal systems pathways and human diseases pathways. All the human diseases pathways were removed in this study. By using KO annotations, we classified the genes into 32 groups based on their participation in KEGG metabolic pathways (Fig 7). In this study, the enriched pathways were ‘metabolism pathways’ (4,475 unigenes), followed by ‘genetic information processing pathways’ (1,939 unigenes).

**Fig 8:**
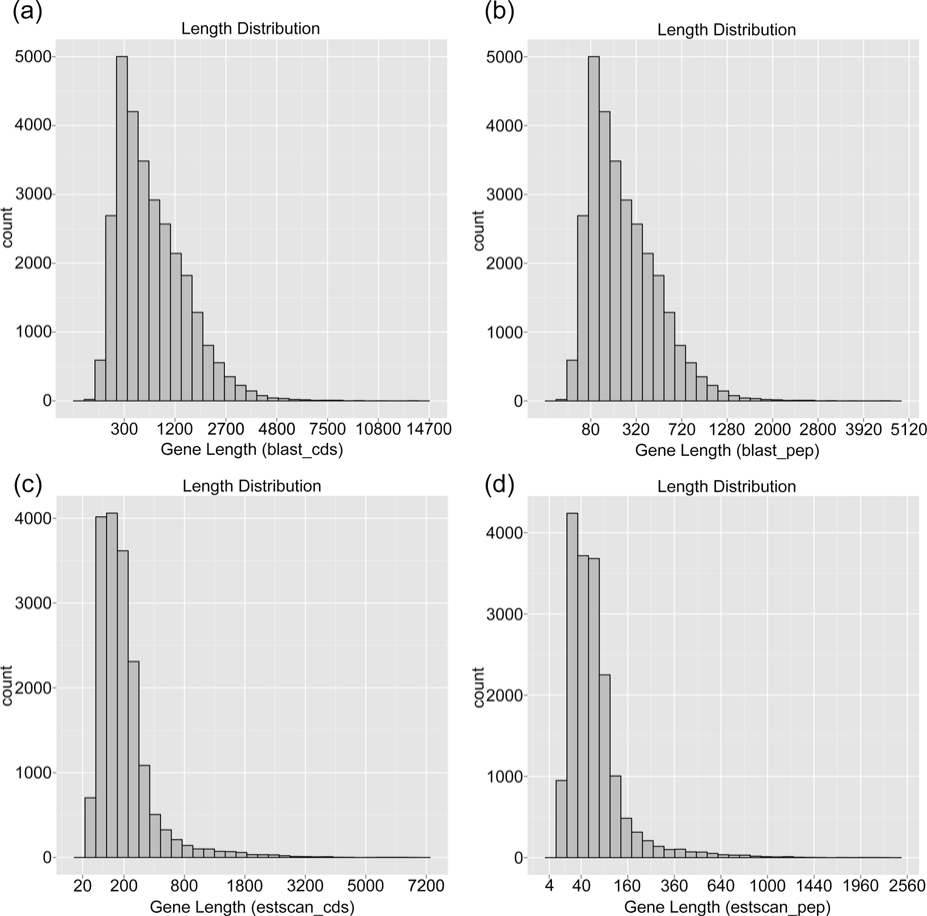
Length distribution of CDS prediction and translation. (a) The length distribution of the predicated CDS sequences using NCBI blast 2.2.28+; (b) The length distribution of the predicated amino acid sequences using NCBI blast 2.2.28+; (c) The length distribution of the predicated CDS sequences using Estscan (3.0.3) software; (d) The length distribution of the predicated amino acid sequences using Estscan (3.0.3) software.

We predicted the protein coding sequence (CDS) and the amino acid sequence of all unigenes using NCBI blast 2.2.28+ and Estscan (3.0.3) software to analyze unigene functions at the protein level. Firstly, the unigenes were searched in the Nr database and Swissprot database, and the corresponding ORF sequence of the unigenes were used to extract the predicted CDS sequence and translated into amino acid sequence with a standard genetic codon table (5' to 3'). The Nr database takes precedence over the Swissprot database. If the unigene did not hit any database, the software Estscan (3.0.3) was employed to predict its ORF which was then converted to CDS sequence and amino acid sequence. Altogether, a total of 29,054 unigenes (about 44.1%) were functionally annotated in the NR and Swissprot databases using NCBI blast 2.2.28+, and 17,554 not-hit unigenes (26.7%) were predicted by the Estscan (3.0.3) software. The length distributions of the predicated CDS sequences and amino acid sequences were displayed in Fig 8. In general, the length distribution of CDS prediction and translation were consistent with unigene assembly results.

### Global analysis of differential gene expression during flower development

To obtain digital gene expression signatures during flower development of the MS and MF plant, we sequenced eleven libraries with three/two replications for flower buds 1 mm and 4 mm in diameter (designated as S1 and S2, F1 and F2). In total, raw reads generated from DGE libraries ranged from 16,249,267 to 21,996,609. After removal of adapter, poly-N and low quality reads, a total of 16,101,543 to 21,795,753 clean reads remained (S3 Table). These trimmed reads were mapped to the reference transcriptome database using RSEM software [25], and the results showed that the total mapped reads ranged from 15,269,622 (94.78%) to 20,699,094 (95.08%) (S3 Table).

Gene expression levels were quantified by RSEM [25] for each sample, and all read counts were normalized to FPKM value. To examine the reliability of data between biological replications, the Pearson’s correlation analysis of gene expression were carried out by SPSS software with transformation of log_10_ (FPKM+1). The Pearson’s correlation coefficients among replications of each sample were all higher than 0.95, indicating satisfactory repeatability (S1 Fig).

**Fig 9:**
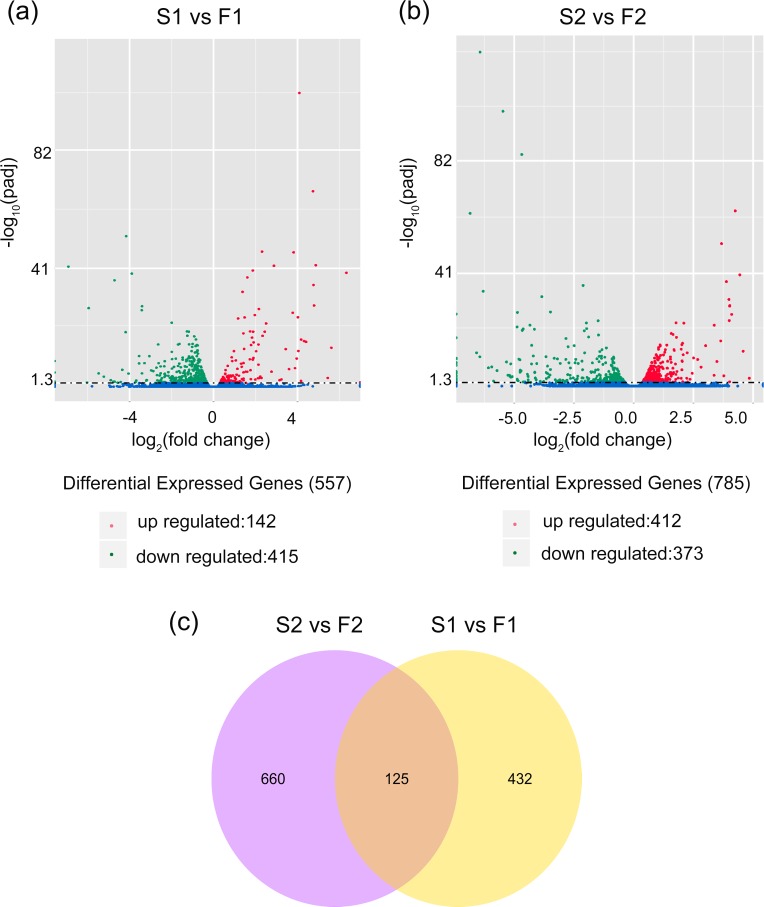
Differentially expressed genes (DEGs) of flower buds from male sterile and male fertile plants. (a) DEGs between S1 (1 mm flower buds of male sterile plants) and F1(1 mm flower buds of male fertile plants); (b) DEGs between S2 (4 mm flower buds of male sterile plants) and F2 (4 mm flower buds of male fertile plants); (c) The Venn diagram showed specifically or commonly expressed DEGs in both development of flower buds. In the volcano figure, scattered dot represented each gene, blue dots indicated that the unigenes with no significant differential expression level, red dots indicated the significantly up-regulated unigenes while the green dots indicated the significantly down-regulated unigenes. In the Venn diagram, the number in the large circle represented total number of specifically expressed DEGs in 1 mm or 4 mm sized flower buds, while the number in the overlapping portion represented commonly expressed DEGs in both 1 mm and 4 mm sized flower buds.

Genes having an adjusted P value < 0.05 found by DESeq were regarded as DEGs. By comparing with F1, 557 transcripts were found to be differentially expressed in the S1 library, which included 142 up-regulated genes and 415 down-regulated genes (Fig 9A). For S2, there were 785 differentially expressed transcripts when compared with F2 library, including 412 up-regulated genes and 373 down-regulated genes (Fig 9B). In addition, 125 transcripts showed significant differential expression levels in both developmental stages (Fig 9C).

**Fig 10:**
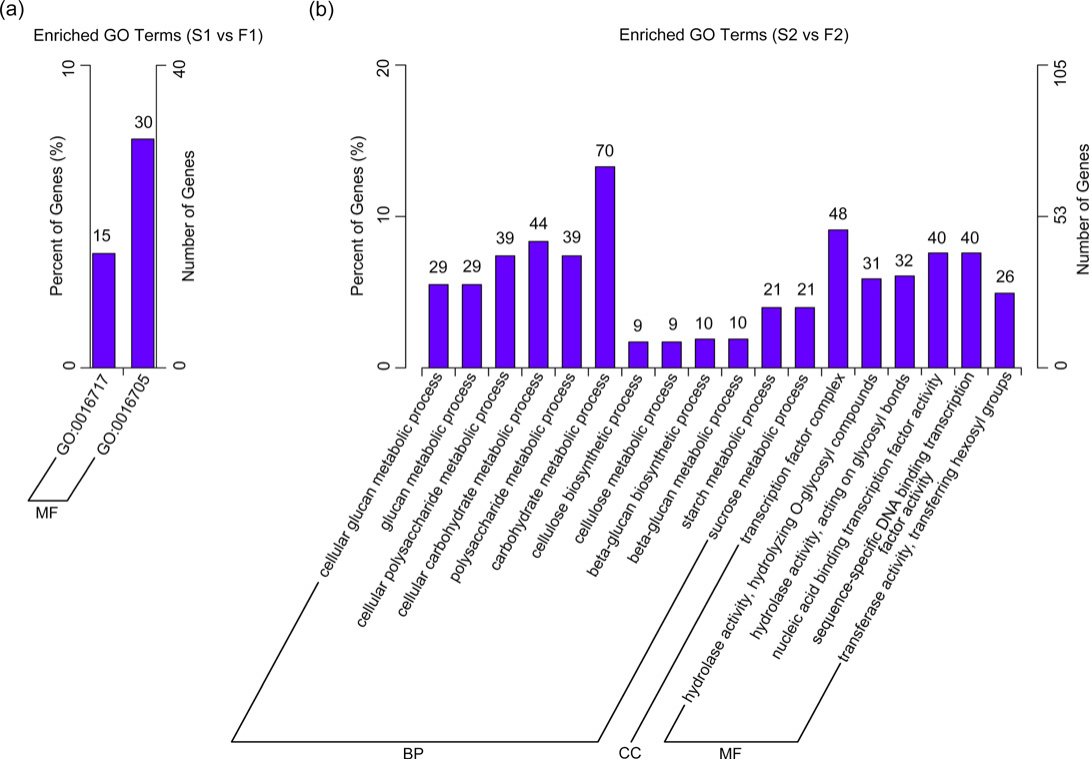
GO term enrichment analysis of differentially expressed genes of flower buds between male sterile and male fertile plants. (a) Enriched GO term between S1 (1 mm flower buds of male sterile plants) and F1 (1 mm flower buds of male fertile plants); (b) Enriched GO term between S2 (4 mm flower buds of male sterile plants) and F2 (4 mm flower buds of male fertile plants). The results were categorized into three major categories (BP: biological process, CC: cellular component, MF: molecular function). The left y-axis represented the percentage of DEGs annotated in this term. The digits above the GO terms represented the number of DEGs annotated in this term (including the sub-term).

To reveal significantly enriched GO terms in DEGs, GO enrichment analysis of functional significance on all DEGs was performed; besides, we also divided these terms into up-regulated and down-regulated groups. The GO term with P value < 0.05 was considered significantly enriched. For the DEGs between S1 and F1, there were two significantly enriched GO terms: oxidoreductase activity acting on paired donors with oxidation of a pair of donors resulting in the reduction of molecular oxygen to two molecules of water (15 genes); and oxidoreductase activity acting on paired donors with incorporation or reduction of molecular oxygen (30 genes). Both GO terms participated in molecular function and most of the DEGs were down-regulated in S1 (Fig 10A). Other significantly down-regulated DEGs were presented in the “lipid metabolic process”, belonging to the biological process (S2 Fig).

Comparing the DEGs between S2 and F2, there were 18 significantly enriched GO terms, including 12 in biological process, 5 in molecular function, and 1 in cellular component. The significantly overrepresented GO terms were ‘carbohydrate metabolic process’ (70 genes), ‘transcription factor complex’ (48 genes), ‘cellular carbohydrate metabolic process’ (44 genes), ‘nucleic acid binding transcription factor activity’ (40 genes), ‘sequence-specific DNA binding transcription factor activity’ (40 genes), ‘cellular polysaccharide metabolic process’ (39 genes), and ‘polysaccharide metabolic process’ (39 genes) (Fig 10B). The down-regulated DEGs in S2 were involved in 10 significantly enriched GO terms. The significantly overrepresented GO terms were “organelle lumen”, “intracellular organelle lumen” and “membrane-enclosed lumen”, all of which belonged to the cellular component category (S3 Fig). The up-regulated DEGs in S2 were showed in 24 significantly enriched GO terms, including 16 in biological process, 2 in cellular component, and 6 in molecular function. The major up-regulated DEGs were seen in “carbohydrate metabolic process”, “cellular carbohydrate metabolic process”, “cellular polysaccharide metabolic process” and “polysaccharide metabolic process” classifications (S4 Fig).

We also conducted KEGG pathway enrichment analysis of DGE to further understand the biological functions of DEGs. The KEGG pathway with corrected P value < 0.05 was considered significantly enriched. The top 20 enriched KEGG pathways corresponding to DEGs detected in both development stages of MS and MF plants were listed in S4 and S5 Tables, respectively. We also conducted the KEGG pathway enrichment analysis of the up-regulated and down-regulated DEGs groups, separately (S6–S9 Tables). For the DEGs between S1 and F1, there were six significantly enriched pathways, and the most significantly over-represented enriched pathways were ‘biosynthesis of unsaturated fatty acids’ (rich factor = 0.3030, P value = 0, 20 genes) and ‘fatty acid metabolism’ (rich factor = 0.1429, P value = 1.94^E-09^, 20 genes) (S4 Table), both of which involved 20 down-regulated DEGs (S6 Table). Other significantly down-regulated DEGs were involved in ‘photosynthesis—antenna proteins’, ‘metabolism of xenobiotics by cytochrome P450’, ‘Drug metabolism—cytochrome P450’, and ‘flavone and flavonol biosynthesis’ pathways (S6 Table). Up-regulated DEGs were mainly found in the ‘Arginine and proline metabolism’ pathway (S7 Table).

Comparing the DEGs between S2 and F2, there were four significantly enriched pathways. The most highly enriched pathway was ‘phenylpropanoid biosynthesis’ (rich factor = 0.0915, P value = 0.0030, 13 genes) for containing most up-regulated DEGs. “Biosynthesis of secondary metabolites” involved the largest number of DEGs (rich factor = 0.0384, P value = 0.0277, 43 genes). Other two significantly enriched pathways were ‘flavonoid biosynthesis’ (rich factor = 0.1304, P value = 0.0304, six genes), and ‘phenylalanine metabolism’ (rich factor = 0.0860, P value = 0.0348, eight genes) (S5 Table), both of which contained both up-regulated and down-regulated DEGs (S8 and S9 Tables).

### MADS-box Genes involved in flower development

**Fig 11:**
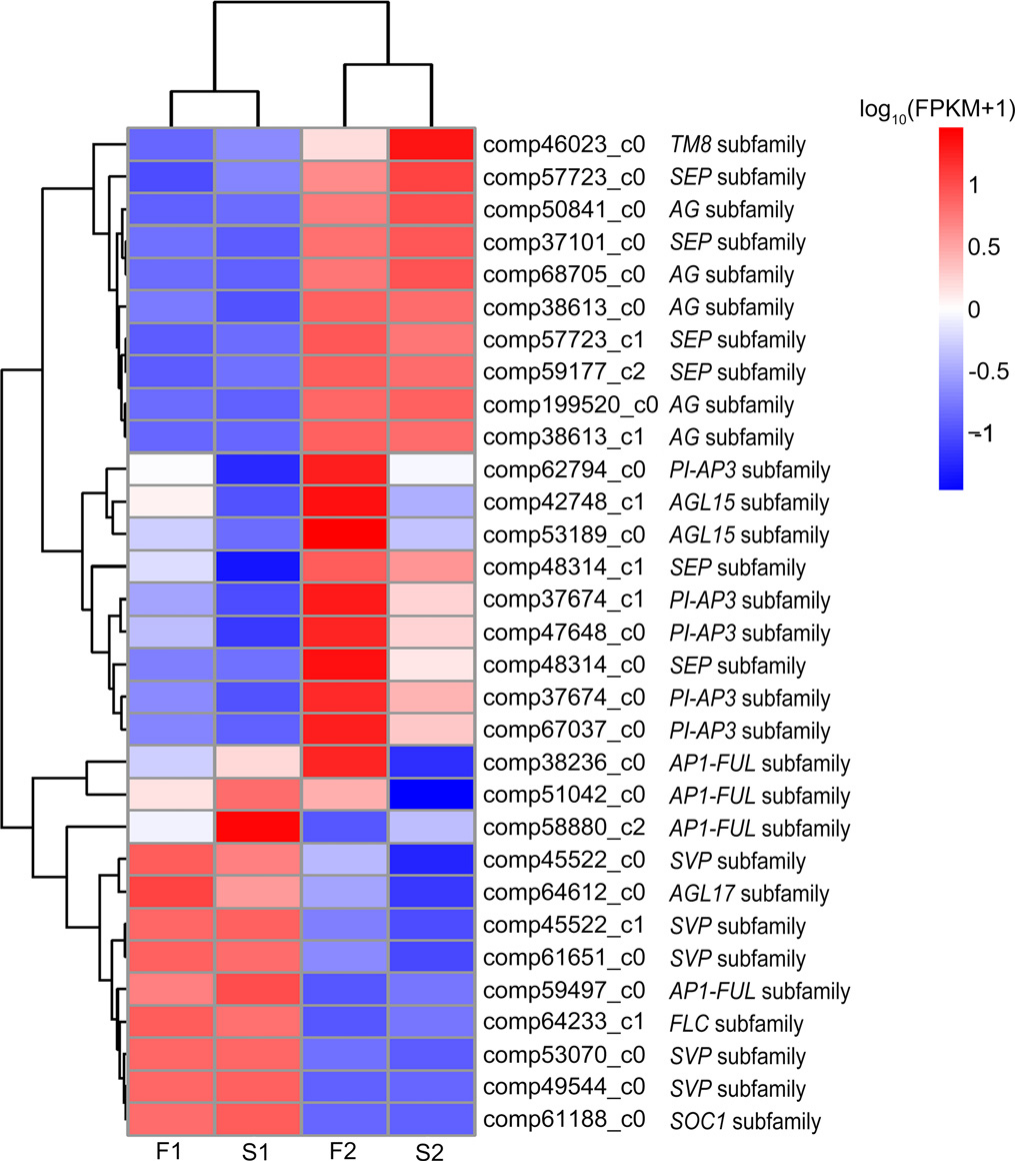
Heat map diagram of expression levels of DEGs annotated in the MADS-box transcription. Data for the relative expression levels of genes were obtained by DGE data after taking log_10_ (FPKM+1). Color from red to blue, indicated that the log_10_ (FPKM+1) values were from large to small, red color indicates high expression level and blue color indicates low expression level.

It has been reported that spontaneous homeotic conversion of floral organs was the underlying cause of the male sterility in this marigold line [11]. So, we specially focused on the MADS-box genes for their regulatory function in floral organs development. The MIKC^c^-type MADS-box genes involved in plant growth and development, especially in specifying the floral organ identity, have been divided into 13 gene subfamilies, termed *AG*, *AGL6*, *AGL12*, *AGL15*, *AGL17*, *AP1-FUL*, *BS*, *FLC*, *PI-AP3*, *SEP*, *SVP*, *SOC1* and *TM8* [37–39]. In our study, 31 unigenes were annotated as the MADS-box transcription factors and displayed substantially different expression levels during the flower development (Fig 11). They could be further classified into 10 subfamilies which were *AG*, *AGL15*, *AGL17*, *AP1-FUL*, *FLC*, *PI-AP3*, *SEP*, *SOC1*, *SVP* and *TM8* (Fig 11).

We looked for the differential expressed genes between S1 and F1, and S2 and F2, respectively. Genes having an adjusted P value < 0.05 found by DESeq were assigned as DEGs. Only one *PI*-like gene (comp62794_c0) showed significantly different expression levels between S1 and F1, and the expression level in S1 was significantly lower than in F1 (adjusted P value = 5.18^E-07^). Between S2 and F2, there were 12 MADS-box unigenes showing significantly different expression levels (Table 2). Compared to the expression level in F2, there were 11 unigenes in S2 with significantly lower expression, including one *PI*-like gene (comp62794_c0), four *AP3*-like genes (comp37674_c0, comp37674_c1, comp67037_c0 and comp47648_c0), two *AP1*-like genes (comp38236_c0 and comp51042_c0), two *AGL15*-like genes (comp42748_c1 and comp53189_c0), one *SEP*-like gene (comp48314_c0), and one *SVP*-like gene (comp45522_c0). By contrast, there was only one *TM8*-like gene (comp46023_c0) expressed higher in S2.

**Table 2:** MADS-box unigenes showing significantly different expression between S2 and F2.

Gene ID	Homologous genes	log_2_^FoldChange^	P value	Adjusted P value
comp62794_c0	*PI*-like gene	-2.0694	3.79E-24	7.42E-21
comp37674_c1	*AP3*-like gene	-1.3483	1.42E-14	1.26E-11
comp53189_c0	*AGL15*-like gene	-3.1943	1.85E-13	1.26E-10
comp47648_c0	*AP3*-like gene	-1.0424	1.42E-08	4.71E-06
comp48314_c0	*SEP*-like gene	-0.87983	7.32E-07	0.0001641
comp67037_c0	*AP3*-like gene	-0.99052	2.84E-06	0.0005529
comp51042_c0	*AP1*-like gene	-1.1712	6.33E-06	0.0011355
comp45522_c0	*SVP*-like gene	-3.1072	4.37E-05	0.0058644
comp38236_c0	*AP1*-like gene	-0.61246	5.62E-05	0.0072351
comp37674_c0	*AP3*-like gene	-0.96502	0.0001062	0.012092
comp46023_c0	*TM8*-like gene	1.3663	0.0002641	0.025272
comp42748_c1	*AGL15*-like gene	-2.1868	0.0004778	0.039343

### Validation of Illumina sequencing results by qRT-PCR

**Fig 12:**
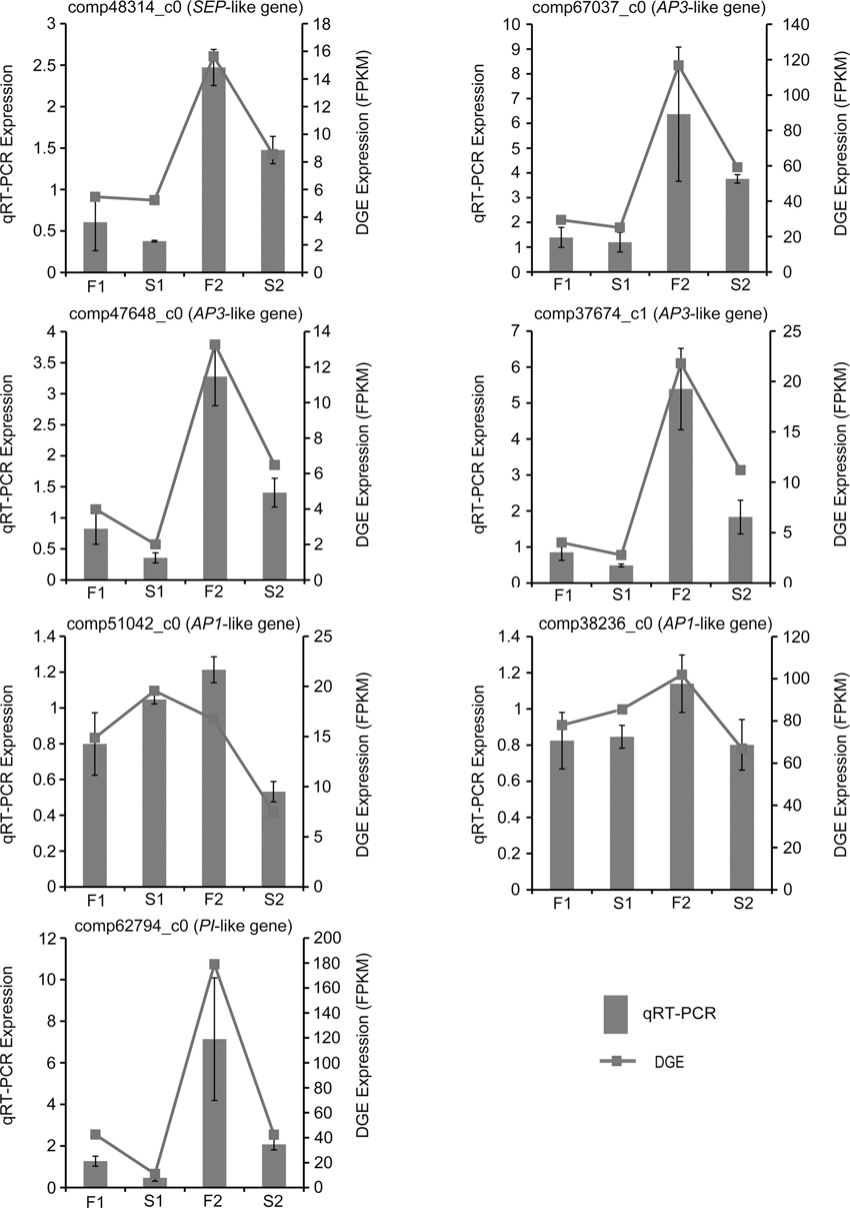
qRT-PCR verifications of seven MADS-box genes. The x axis represented four samples. S1: 1 mm flower buds of male sterile plants, F1: 1 mm flower buds of male fertile plants, S2: 4 mm flower buds of male sterile plants, F2: 4 mm flower buds of male fertile plants. The Left y axis represented the relative expression level by qRT-PCR. The right y axis is the FPKM value by DGE analysis.

**Fig 13:**
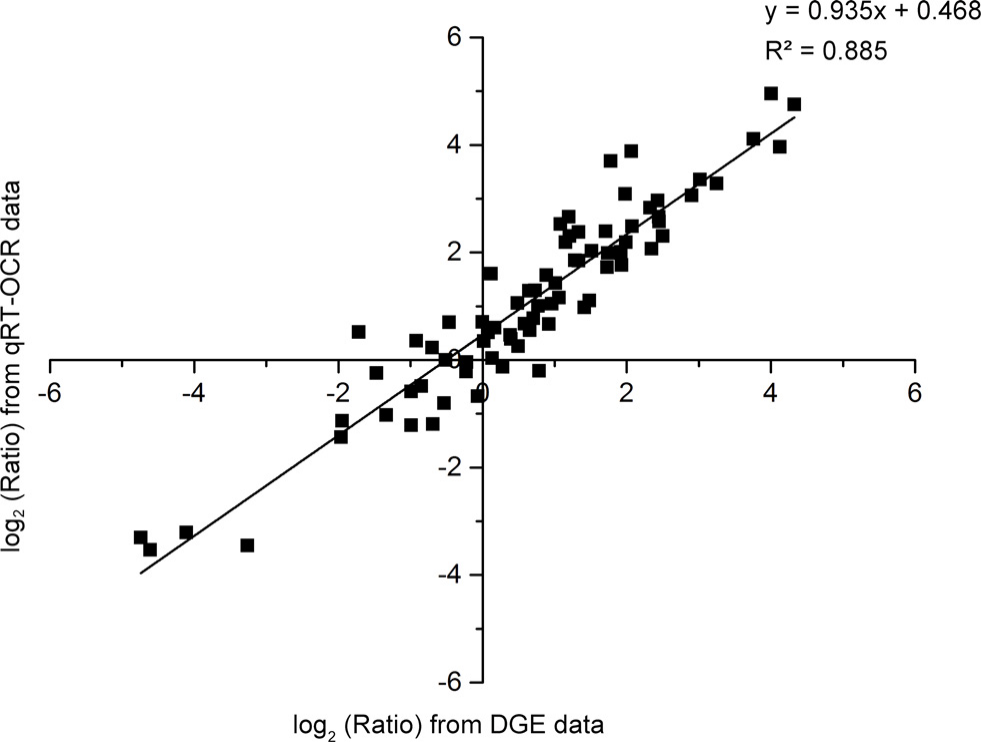
Linear regression analysis of the fold change of the gene expression ratios between DEG sequencing and qRT-PCR. 26 unigenes were selected for quantitative real-time PCR analysis to confirm the accuracy and reproducibility of the Illumina expression profiles using the same RNA samples that were used for DGE sequencing. The relative expression levels of the genes were calculated using the 2^−ΔΔCt^ method in qRT-PCR analysis. The DGE sequencing data were represented by the FPKM value of samples. Scatterplots were generated by the log_2_ expression ratios from DGE sequencing data (x-axis) and qRT-PCR data (y-axis).

To confirm the accuracy and reproducibility of the Illumina expression profiles, qRT-PCR analysis was performed to analyze the expression levels of seven MADS-box genes (Fig 12) and 19 randomly selected unigenes. The expression levels of each gene in S1, F1, S2, and F2 were measured through qRT-PCR and compared with its abundance from DGE sequencing data. The relative expression levels of the genes were calculated using the 2^−ΔΔCt^ method in qRT-PCR analysis. The DGE sequencing data were represented by the FPKM value of samples. Linear regression analysis showed significantly positive correlation (R^2^ = 0.885) between DGE sequencing and qRT-PCR in the fold change of the gene expression ratios (Fig 13), suggesting that the expression of the 26 unigenes revealed by qRT-PCR agreed well with the DGE analysis, thus confirmed the Illumina expression profiles analysis.

## Discussion

So far, the lack of genome and transcriptome data has greatly restricted molecular studies in *T*. *erecta*. Here, we adopted the Illumina sequencing technology for de novo reference transcriptome assembly using flowering buds of *T*. *erecta*. A total of 87,473,431 clean reads were generated by Illumina HiSeq 2000, and 65,857 unigenes were assembled using the Trinity software, including many transcripts in the floral organ development. Among the Nr, Nt, Pfam, KOG, Swiss-Pro, KO and GO databases, 34,176 unigenes (51.89%) were annotated in at least one database and 3,481 unigenes (5.28%) were annotated in all databases, demonstrating that a large proportion of unigenes have clear descriptions of their functions. Through gene functional annotation, we could not only assess the functions of the unigenes, but get an insight into the putative conserved domains, gene ontology terms, and potential metabolic pathways [40]. This work is the first attempt to sequence and assemble a reference transcriptome in *T*. *erecta* using Illumina sequencing technology. Our results will provide a valuable resource for future genomic studies on *T*. *erecta* and other Asteraceae species, especially in flower organ development and/or differentiation. However, there were still nearly half of the unigenes cannot be annotated in any of the seven databases. Similar phenomena were also reported in other Asteraceae plants, such as *Carthamus tinctorius* [40], *Gerbera hybrida* [41], and *Chrysanthemum nankingense* [42]. The reason may lie in the uniqueness of unigenes in Asteraceae family and further studies are needed to understand the biological functions of those non-annotated unigenes.

DGE analysis is a powerful tool to identify and quantify gene expression on the whole genome level. When compared with traditional technologies, such as RDA (representational difference analysis), SSH (suppression subtractive hybridization), cDNA-AFLP (DNA amplified fragment length polymorphism) and RFDD-PCR (restriction fragment differential display PCR), DGE, a sequencing based method, could provide comprehensive sequencing data for studying differentially expressed genes [13]. Recently, transcriptome and DGE techniques have been successfully utilized to study the molecular mechanism of sterility and to identify the candidate regulators or genes responsible for anther and pollen development in many plant species [23–27]. In this study, 1 mm and 4 mm sized flower buds of MS and MF plants of *T*. *erecta* were designated for DGE analysis to profile the differences at the transcriptional level and identify candidate genes associated with male sterility. According to the DGE results, we detected 557 transcripts with significantly different expression levels between S1 and F1, and 785 transcripts between S2 and F2. Most of these differentially expressed genes were annotated in the public databases. These annotated genes might be candidates causing male sterility in *T*. *erecta* and could provide an invaluable resource to identify genes involved in flower development. To further understand the biological functions of DEGs, GO term and KEGG pathway enrichment analysis were employed to analyze the DEGs. These DGE analysis results will provide a better understanding in the molecular mechanism of male sterility in *T*. *erecta*.

The male sterility of *T*. *erecta* was not due to the failure of anther or pollen development, but as a result of the male organ abnormality caused by homeotic conversion of floral organs [11]. Most floral organ determined genes have been categorized into the family of MADS-box genes [12, 43–45] and have been further grouped into five different classes (A, B, C, D and E) based on their biological functions [38]. They were considered critical to define the differentiation of four whorl floral organs, and loss-of-function of any class of MADS-box genes may result in homeotic conversion of floral organs. According to the ABCDE model of flower organ development, the class A genes (*AP1*, *CAL* and *AP2*) specify the sepal identity in the first whorl; the class A, B (*AP3* and *PI*) and E (*AGL3* and *SEP*) genes collectively control the petal identity in the second whorl; the class B, C (*AG*) and E genes all together control the stamen identity in the third whorl; the class C and E genes combined to determine the formation of the carpel in the fourth whorl; the class D (*SHP1* and *SHP2*, *AGL11* and *AGL13*) and E genes jointly determine the formation of the ovule [46–50].

In our study, only one *PI-*like unigene had significant differential expression levels at the beginning of floret differentiation between S1 and F1, and its expression level in S1 was significantly lower than in F1 (Fig 12). *PI*-like genes belonged to B class genes, and loss-of-function of B class genes produced homeotic phenotypes in which the second whorl organs developed into sepaloid structures, and the third whorl organs developed into carpeloid structures [51, 52]. This conclusion was confirmed by many other researches in the past decades. The co-suppression of *FBP1*, a *PI*-like gene in petunia, resulted in homeotic conversions of petals toward sepals and stamens toward carpels [53]. *MdPI*, identified in apple (*Malus domestica*), not only had a function of floral organ determination but played a role in apple parthenocarpy [54]. In grapevine (*Vitis vinifera*), the mutants showing abnormal petal / stamen structures had low expression level of *VvMADS9*, an orthologue of *PI* gene [55]. In California poppy (*Eschscholzia californica*), the truncation of highly conserved PI motif in SEI-1 protein affected the formation of higher order complexes causing homeotic conversions [56]. Although the regulatory and protein-protein interactions of B-class factors have undergone changes during evolution, they still have conserved functions among flowering plants [12, 37, 57–60]. Thus, it seems likely that the *PI*-like gene might be the promising candidate gene conferring homeotic conversion in *T*. *erecta*.

Based on the DEGs results, 12 unigenes belonging to MADS-box family showed significantly different expression levels between S2 and F2 which contain florets at various differentiation stages, including the ones in the center that just began differentiation, and the ones on the peripheral that had already completed differentiation (Fig 12, Table 2). Compared to the expression level of F2, 11 unigenes expressed significantly lower in S2, including one *PI*-like gene, four *AP3*-like genes, two *AP1*-like genes, two *AGL15*-like genes, one *SEP*-like gene, and one *SVP*-like gene. By contrast, there was one *TM8*-like gene expressing higher in S2. The *SVP*-like, *AGL15*-like and *TM8*-like genes were reported to be involved in floral transition and to determine flowering time [61–63]. The *PI*-like, *AP3*-like, *AP1*-like, and *SEP*-like genes were floral organ identity genes, belonging to the B-, B-, A-, and E-class floral homeotic genes, respectively. Based on the ABCDE model, AP3 and PI proteins are functional partners interacting with each other to form obligate heterodimers for DNA binding in vitro and to regulate gene expression by binding to the CArG motif of their promoters. A complex comprising of AP3/PI/SEP3/AP1 was postulated to specify petals formation and a complex comprising of AP3/PI/SEP3/AG specify stamen development [64].The decreased expression levels of the A-, B-, and E-class genes might influence the formation and function of the heterodimers or high order complex in MS plants. We hypothesized that male sterility of *T*. *erecta* might be related to the suppressed expression of *PI*-like gene at the beginning of floret differentiation, which could affect the formation of PI/AP3 heterodimer and furtherly influence the quaternary complexes of AP3/PI/SEP3/AP1 and AP3/PI/SEP3/AG, leading to the absence of normal petal and stamen organs in MS *T*. *erecta*.

## Supporting Information

S1 Fig Pearson’s correlation analysis of gene expression between samples. F1-1, F1-2, F1-3 and F2-1, F2-2, F2-3 were different replications of F1 (1 mm flower buds of male fertile plants) and F2 (4 mm flower buds of male fertile plants), respectively. S1-1, S1-2, and S2-1, S2-2, S2-3 were different replications of S1 (1 mm flower buds of male sterile plants) and S2 (4 mm flower buds of male sterile plants), respectively. The number represented the Pearson’s correlation analysis of gene expression between samples, the value ranges from 0 to 1. A high value between the biological samples indicated that the samples have good repeatability.
(TIF)

S2 Fig GO term enrichment analysis of down-regulated DEGs of 1 mm flower buds between male sterile and male fertile plants. BP: biological process, MF: molecular function. The x-axis represents the categories of GO terms, the left y-axis represents the percentage of DEGs annotated in this term, and the digits above the GO terms represent the number of DEGs annotated in this term.
(TIF)

S3 Fig GO term enrichment analysis of down-regulated DEGs of 4 mm flower buds between male sterile and male fertile plants. CC: cellular component, MF: molecular function. The x-axis represents the categories of GO terms, the left y-axis represents the percentage of DEGs annotated in this term, and the digits above the GO terms represent the number of DEGs annotated in this term.
(TIF)

S4 Fig GO term enrichment analysis of up-regulated DEGs of 4 mm flower buds between male sterile and male fertile plants. BP: biological process, CC: cellular component, MF: molecular function. The x-axis represents the categories of GO terms, the left y-axis represents the percentage of DEGs annotated in this term, and the digits above the GO terms represent the number of DEGs annotated in this term.
(TIF)

S1 Table Primers of the selected unigenes for qRT-PCR. (DOCX)

S2 Table Length distribution of unigenes and transcripts. (DOCX)

S3 Table Summary of the sequencing data quality of the eleven digital gene expression profiles. (DOCX)

S4 Table The top 20 enriched KEGG pathways of differentially expressed genes of 1 mm flower buds between male sterile and male fertile plants. (DOCX)

S5 Table The top 20 enriched KEGG pathways of differentially expressed genes of 4 mm flower buds between male sterile and male fertile plants. (DOCX)

S6 Table The top 20 enriched KEGG pathways of down-regulated DEGs of 1 mm flower buds between male sterile and male fertile plants. (DOCX)

S7 Table The top 16 enriched KEGG pathways of up-regulated DEGs of 1 mm flower buds between male sterile and male fertile plants. (DOCX)

S8 Table The top 20 enriched KEGG pathways of down-regulated DEGs of 4 mm flower buds between male sterile and male fertile plants. (DOCX)

S9 Table The top 20 enriched KEGG pathways of up-regulated DEGs s of 4 mm flower buds between male sterile and male fertile plants. (DOCX)
