# Supplementary figures and images for: Transcriptomic Analysis of Differentially Expressed Genes during Flower Organ Development in Genetic Male Sterile and Male Fertile Tagetes erecta by Digital Gene-Expression Profiling
Source: PLoS One. 2016 Mar 3;11(3):e0150892. doi: 10.1371/journal.pone.0150892 (PMC4777371; doi:10.1371/journal.pone.0150892)

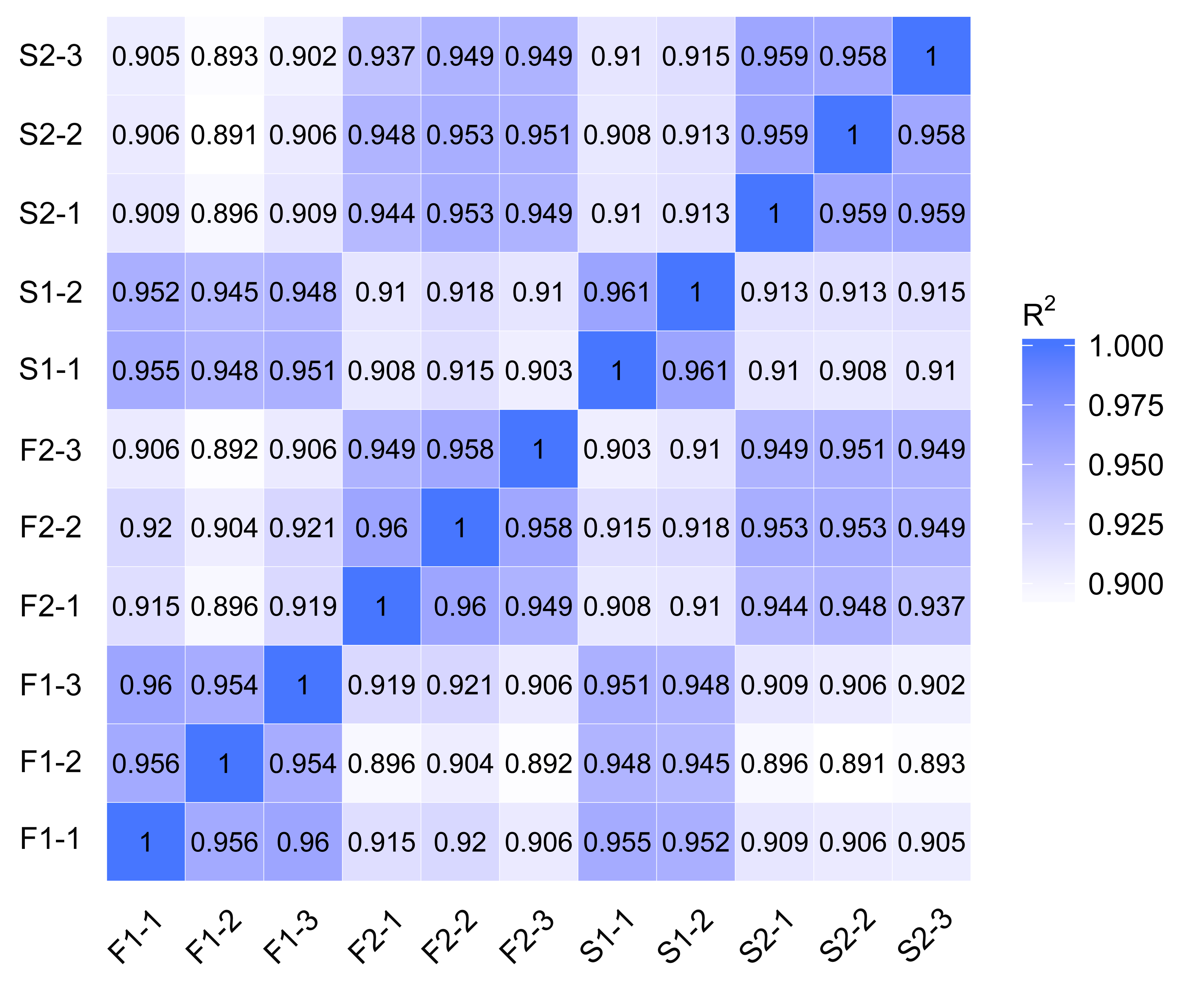

Supplement: S1 Fig — F1-1, F1-2, F1-3 and F2-1, F2-2, F2-3 were different replications of F1 (1 mm flower buds of male fertile plants) and F2 (4 mm flower buds of male fertile plants), respectively. S1-1, S1-2, and S2-1, S2-2, S2-3 were different replications of S1 (1 mm flower buds of male sterile plants) and S2 (4 mm flower buds of male sterile plants), respectively. The number represented the Pearson’s correlation analysis of gene expression between samples, the value ranges from 0 to 1. A high value between the biological samples indicated that the samples have good repeatability. (TIF) [file pone.0150892.s001.tif]

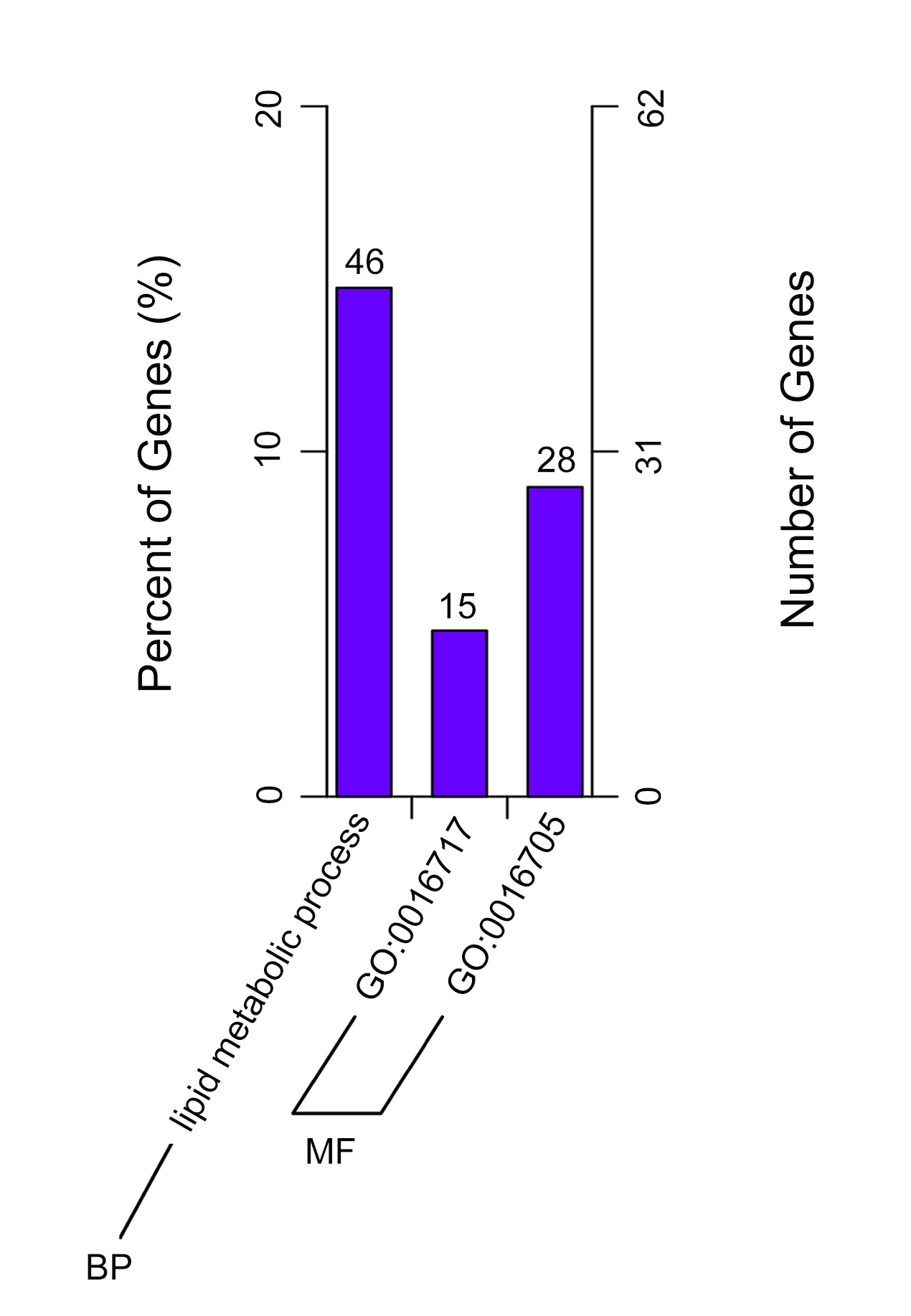

Supplement: S2 Fig — BP: biological process, MF: molecular function. The x-axis represents the categories of GO terms, the left y-axis represents the percentage of DEGs annotated in this term, and the digits above the GO terms represent the number of DEGs annotated in this term. (TIF) [file pone.0150892.s002.tif]

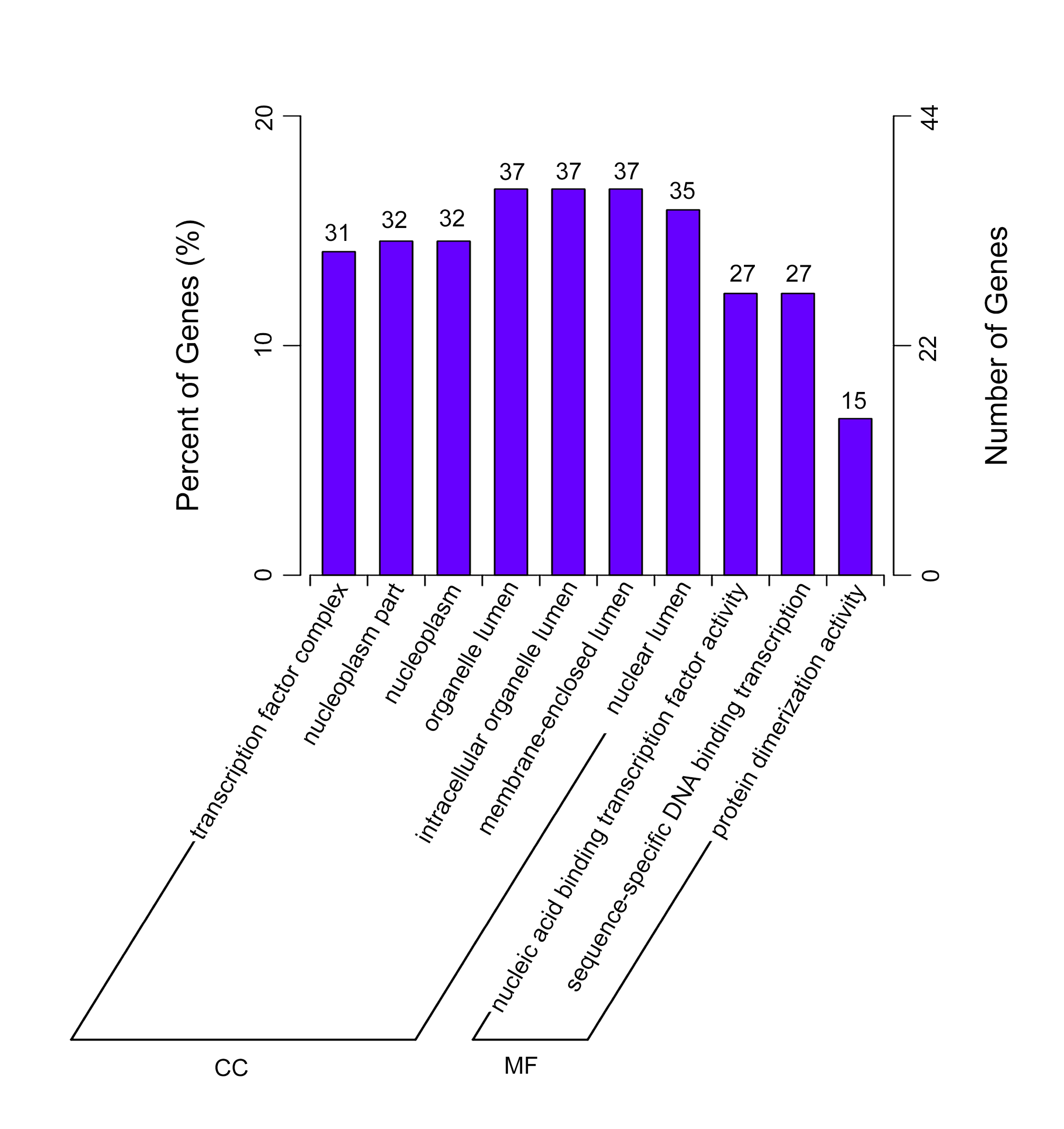

Supplement: S3 Fig — CC: cellular component, MF: molecular function. The x-axis represents the categories of GO terms, the left y-axis represents the percentage of DEGs annotated in this term, and the digits above the GO terms represent the number of DEGs annotated in this term. (TIF) [file pone.0150892.s003.tif]

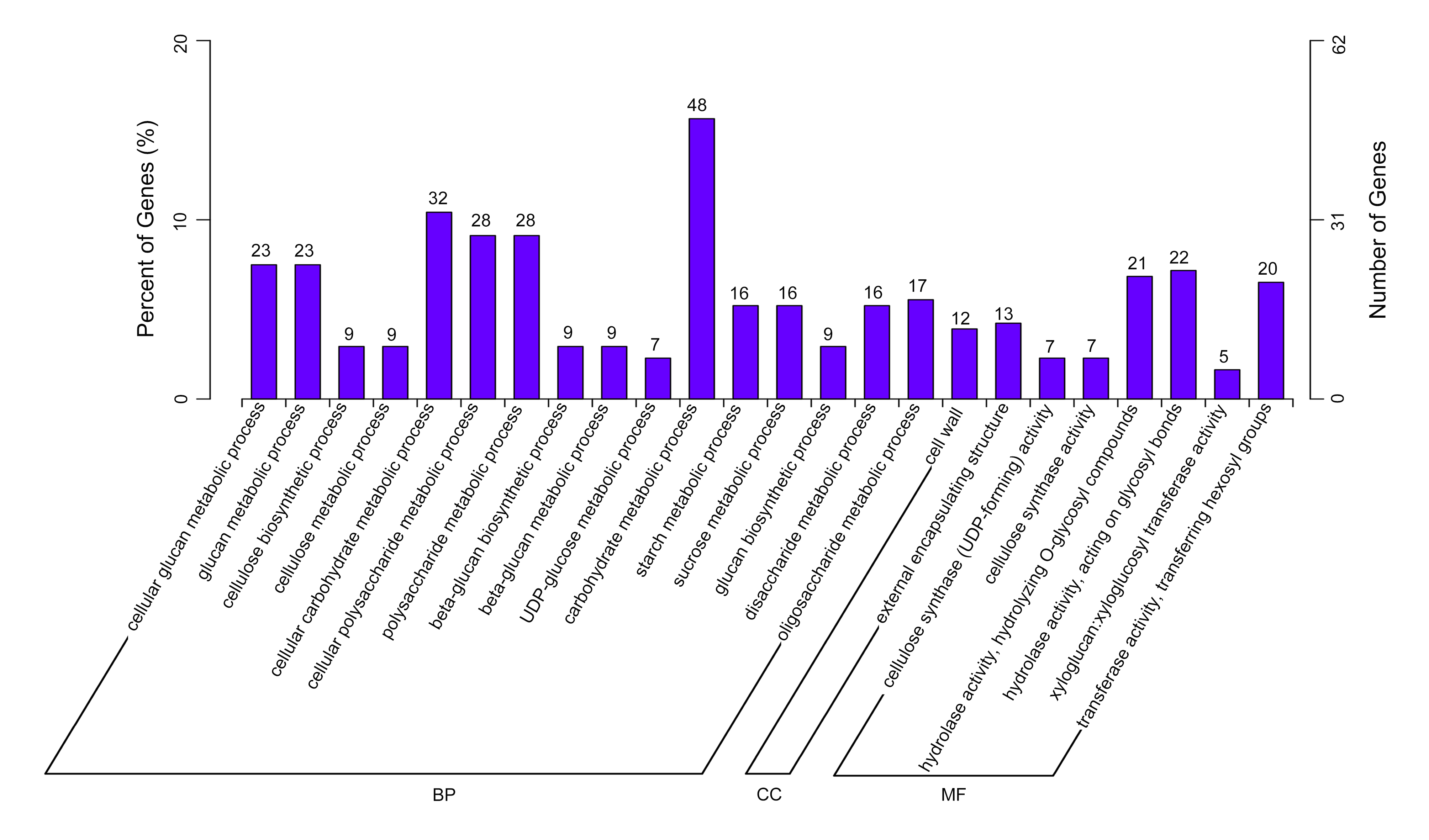

Supplement: S4 Fig — BP: biological process, CC: cellular component, MF: molecular function. The x-axis represents the categories of GO terms, the left y-axis represents the percentage of DEGs annotated in this term, and the digits above the GO terms represent the number of DEGs annotated in this term. (TIF) [file pone.0150892.s004.tif]
