# Supplementary material for: Transcriptomic Analysis of Differentially Expressed Genes during Flower Organ Development in Genetic Male Sterile and Male Fertile Tagetes erecta by Digital Gene-Expression Profiling
Source: PLoS One. 2016 Mar 3;11(3):e0150892. doi: 10.1371/journal.pone.0150892 (PMC4777371; doi:10.1371/journal.pone.0150892)
Supplement: S1 Table — (DOCX) [file pone.0150892.s005.docx]

**S1 Table. Primers of the selected unigenes for the qRT-PCR**

| **Primer** | **Forward primer Sequence (5'to3')** | **Reverse primer Sequence (5'to3')** |
| --- | --- | --- |
| comp34310_c0 | CCACCTCTCCTATCACTCTTCCTAC | TGTTTCCTCTTTCACTTTCTCTCAG |
| comp38537_c0 | GGAGTGGCGTGAAAACGAGCA | CGAGGGCGGAAACTAAATCATC |
| comp37674_c0 | TACTGTTGGCTTATTCATAACCTCG | CAGTATCTGAAAACACAAAACAAAACC |
| comp38236_c0 | GTCAACCGGCAACAATAATACCAG | CATATGCATCCATCTCCATATACCAC |
| comp47648_c0 | ATGGTGGAGAATGAAGGATGCTAC | CATGATTTAAAAACTCAATCGAGACG |
| comp50447_c0 | GATGGAAAGTGGATGTGAGAATGTG | TACTCCAATGGCGGATCAAACTTAC |
| comp51042_c0 | GATACCTTCATTTCATCTTGGCATG | CTTCTCTTGGGTTCTCTTCTACTTCC |
| comp51656_c0 | GAGCACCGCTAAGAGTTTTGACC | GAAAGTTACCAGGCGAAAAGAAGTC |
| comp52554_c0 | GTCAATCTGAAAAACCAGATGTGTC | ATAGAGCAAAACCCCACGAGGAG |
| comp55839_c1 | GCTCCTCCCAATACTATTACCAG | GTTACACACAAAACCCACACATC |
| comp56812_c0 | GCGAAAACGAGTGAAAGAGTGTG | TCACTGATGTCGTGTCCGTTCC |
| comp58128_c0 | GGCATACTTGTTGCTGAGGGTT | ACTGCCGTCTTCGTTCCAATG |
| comp59511_c0 | AGACTGTCCCATTCAGACGCTC | AACACGCCAACACACGGATG |
| comp60520_c0 | ATCCCATCTCTTCTCGTGCTG | TCGGTCAATCTCCTTTTCGTG |
| comp62673_c1 | CTCTGCCTCTTCTATCTTCCCTATC | CTTCTTCATCCAGTAATGGCGTC |
| comp62794_c0 | GAAATCCCTAAAATGATGAGGAAGC | ATCGGTTGAACACGGAACGAAAAG |
| comp64004_c1 | GTATCAAGAACGCAGCACCACTC | ACCTCCTACTGTATCCATCGCTC |
| comp64375_c0 | GCTTAGAGTTGCGAAGACGGTTG | ATTTCGTCCAAGTTCGGATGAGC |
| comp64808_c1 | AACACGACCCGTTTAGCACCTC | ATTCACAACCCGCCTACCACAG |
| comp67037_c0 | CAAGCTCAAAGTGATCGGTAATAAG | ATATGTGGTGGGTATCCATACAAAG |
| comp68452_c0 | CAATGGGGAAGGAGTATGTGGTC | GGGCTTTCAAGTCTTATCAATCG |
| comp68461_c0 | GCCCATCTACACTCTCATTCACAC | GGATGGCAATAATACCTCTTCTTCA |
| comp71322_c0 | GAAGTGGCATTTGGCTCTGTAAC | TGCTTTGACTGACGGAGATTTTAG |
| comp48314_c0 | TTGCTAGAAAGGCAGCTCGATAC | CTTGTGGAAACCAGTGGAATGAG |
| comp65196_c0 | CAGTGTTAGAGAAGGTAGAGGGCAT | AATCATCGTATTGTGACCGCAGG |
| comp66824_c0 | AGACGCTGGCTGTGATGCTTG | GATTCGTTTCTGTTTCCTTCATACC |
| WSJ-RT-ACT-F | GGGAAATGAATGCCAAAGCCAAG | AAGACTTCACAACCACTCTCCAACT |
