# Supplementary material for: Transcriptomic Analysis of Differentially Expressed Genes during Flower Organ Development in Genetic Male Sterile and Male Fertile Tagetes erecta by Digital Gene-Expression Profiling
Source: PLoS One. 2016 Mar 3;11(3):e0150892. doi: 10.1371/journal.pone.0150892 (PMC4777371; doi:10.1371/journal.pone.0150892)
Supplement: S2 Table — (DOCX) [file pone.0150892.s006.docx]

**S2 Table. Length distribution of unigenes and transcripts**

| **Nucleotide length** | **Number of transcripts** | **Number of unigenes** |
| --- | --- | --- |
| 200-500 bp | 46,772 | 37,921 |
| 500-1k bp | 26,055 | 12,661 |
| 1k-2k bp | 31,952 | 9,541 |
| >2k bp | 24,158 | 5,734 |
| Min Length | 201 | 201 |
| Mean Length | 1,188 | 777 |
| Median Length | 806 | 413 |
| Max Length | 13,680 | 13,680 |
| N50 | 1,928 | 1,379 |
| N90 | 523 | 296 |
