# Supplementary material for: Transcriptomic Analysis of Differentially Expressed Genes during Flower Organ Development in Genetic Male Sterile and Male Fertile Tagetes erecta by Digital Gene-Expression Profiling
Source: PLoS One. 2016 Mar 3;11(3):e0150892. doi: 10.1371/journal.pone.0150892 (PMC4777371; doi:10.1371/journal.pone.0150892)
Supplement: S3 Table — (DOCX) [file pone.0150892.s007.docx]

**S3 Table. Summary of the sequencing data quality of the eleven digital gene expression profiles**

| **Sample** | **Raw Reads** | **Clean Reads** | **Clean Bases** | **Total mapped to ref** | **Error (%)** | **Q20 (%)** | **Q30 (%)** | **GC Content (%)** |
| --- | --- | --- | --- | --- | --- | --- | --- | --- |
| F1-1 | 17,957,580 | 17,791,726 | 1.78G | 16,913,740(95.07%) | 0.03 | 97.51 | 91.82 | 42.26 |
| F1-2 | 16,249,267 | 16,101,543 | 1.61G | 15,269,622(94.83%) | 0.03 | 97.48 | 91.75 | 42.23 |
| F1-3 | 17,155,434 | 17,025,981 | 1.7G | 16,181,171(95.04%) | 0.03 | 97.44 | 91.58 | 42.29 |
| F2-1 | 17,320,599 | 17,136,762 | 1.71G | 16,257,691(94.87%) | 0.03 | 97.52 | 91.8 | 42.18 |
| F2-2 | 21,996,609 | 21,795,753 | 2.18G | 20,699,094(94.97%) | 0.03 | 97.49 | 91.72 | 41.95 |
| F2-3 | 16,801,953 | 16,652,194 | 1.67G | 15,782,161(94.78%) | 0.03 | 97.55 | 91.89 | 41.98 |
| S1-2 | 19,348,902 | 19,160,486 | 1.92G | 18,203,656(95.01%) | 0.03 | 97.49 | 91.74 | 42.17 |
| S1-3 | 16,467,980 | 16,297,812 | 1.63G | 15,485,497(95.02%) | 0.03 | 97.49 | 91.76 | 42.3 |
| S2-1 | 20,045,606 | 19,865,613 | 1.99G | 18,858,569(94.93%) | 0.03 | 97.41 | 91.5 | 42.17 |
| S2-2 | 17,937,325 | 17,796,865 | 1.78G | 16,920,511(95.08%) | 0.03 | 97.53 | 91.81 | 42.26 |
| S2-3 | 18,336,196 | 18,143,709 | 1.81G | 17,219,171(94.90%) | 0.03 | 97.54 | 91.86 | 42.03 |
