# Supplementary material for: Transcriptomic Analysis of Differentially Expressed Genes during Flower Organ Development in Genetic Male Sterile and Male Fertile Tagetes erecta by Digital Gene-Expression Profiling
Source: PLoS One. 2016 Mar 3;11(3):e0150892. doi: 10.1371/journal.pone.0150892 (PMC4777371; doi:10.1371/journal.pone.0150892)
Supplement: S4 Table — (DOCX) [file pone.0150892.s008.docx]

**S4 Table. The top 20 enriched KEGG pathways of differentially expressed genes of 1 mm flower buds between male sterile and male fertile plants**

| **Pathway term** | **Rich factor** | **Correct P value** | **Gene number** |
| --- | --- | --- | --- |
| Biosynthesis of unsaturated fatty acids | 0.303030303 | 0 | 20 |
| Fatty acid metabolism | 0.142857143 | 1.94E-09 | 20 |
| Photosynthesis - antenna proteins | 0.294117647 | 0.001356 | 5 |
| Metabolism of xenobiotics by cytochrome P450 | 0.155555556 | 0.001649 | 7 |
| Drug metabolism - cytochrome P450 | 0.155555556 | 0.001649 | 7 |
| Flavone and flavonol biosynthesis | 0.222222222 | 0.015283 | 4 |
| Arginine and proline metabolism | 0.08045977 | 0.070032 | 7 |
| Glutathione metabolism | 0.07 | 0.132452 | 7 |
| Cysteine and methionine metabolism | 0.067961165 | 0.137994 | 7 |
| Nitrogen metabolism | 0.107142857 | 0.361267 | 3 |
| Amino sugar and nucleotide sugar metabolism | 0.050314465 | 0.361267 | 8 |
| Alpha-Linolenic acid metabolism | 0.06557377 | 0.632691 | 4 |
| Flavonoid biosynthesis | 0.065217391 | 1 | 3 |
| Sphingolipid metabolism | 0.090909091 | 1 | 2 |
| Stilbenoid, diarylheptanoid and gingerol biosynthesis | 0.058823529 | 1 | 3 |
| Alanine, aspartate and glutamate metabolism | 0.058823529 | 1 | 3 |
| Glycosphingolipid biosynthesis - ganglio series | 0.2 | 1 | 1 |
| Plant hormone signal transduction | 0.034090909 | 1 | 9 |
| Benzoate degradation | 0.166666667 | 1 | 1 |
| Photosynthesis | 0.050847458 | 1 | 3 |
