# Supplementary material for: Transcriptomic Analysis of Differentially Expressed Genes during Flower Organ Development in Genetic Male Sterile and Male Fertile Tagetes erecta by Digital Gene-Expression Profiling
Source: PLoS One. 2016 Mar 3;11(3):e0150892. doi: 10.1371/journal.pone.0150892 (PMC4777371; doi:10.1371/journal.pone.0150892)
Supplement: S5 Table — (DOCX) [file pone.0150892.s009.docx]

**S5 Table. The top 20 enriched KEGG pathways of differentially expressed genes of 4 mm flower buds between male sterile and male fertile plants**

| **Pathway term** | **Rich factor** | **Correct P value** | **Gene number** |
| --- | --- | --- | --- |
| Phenylpropanoid biosynthesis | 0.091549296 | 0.003039 | 13 |
| Biosynthesis of secondary metabolites | 0.038358608 | 0.027723 | 43 |
| Flavonoid biosynthesis | 0.130434783 | 0.030351 | 6 |
| Phenylalanine metabolism | 0.086021505 | 0.048345 | 8 |
| Plant hormone signal transduction | 0.053030303 | 0.08616 | 14 |
| Fatty acid elongation | 0.111111111 | 0.092172 | 5 |
| Degradation of aromatic compounds | 0.2 | 0.103145 | 3 |
| Metabolism of xenobiotics by cytochrome P450 | 0.088888889 | 0.345236 | 4 |
| Drug metabolism - cytochrome P450 | 0.088888889 | 0.345236 | 4 |
| Stilbenoid, diarylheptanoid and gingerol biosynthesis | 0.078431373 | 0.415255 | 4 |
| Cysteine and methionine metabolism | 0.058252427 | 0.415255 | 6 |
| Naphthalene degradation | 0.166666667 | 0.415255 | 2 |
| Cutin, suberine and wax biosynthesis | 0.09375 | 0.458822 | 3 |
| Streptomycin biosynthesis | 0.133333333 | 0.542056 | 2 |
| Retinol metabolism | 0.111111111 | 0.686647 | 2 |
| Galactose metabolism | 0.059701493 | 0.686647 | 4 |
| Cyanoamino acid metabolism | 0.065217391 | 0.858284 | 3 |
| Chloroalkane and chloroalkene degradation | 0.086956522 | 0.905293 | 2 |
| Nitrogen metabolism | 0.071428571 | 1 | 2 |
| Arginine and proline metabolism | 0.045977011 | 1 | 4 |
