# Supplementary material for: Transcriptomic Analysis of Differentially Expressed Genes during Flower Organ Development in Genetic Male Sterile and Male Fertile Tagetes erecta by Digital Gene-Expression Profiling
Source: PLoS One. 2016 Mar 3;11(3):e0150892. doi: 10.1371/journal.pone.0150892 (PMC4777371; doi:10.1371/journal.pone.0150892)
Supplement: S6 Table — (DOCX) [file pone.0150892.s010.docx]

**S6 Table. The top 20 enriched KEGG pathways of down-regulated DEGs of 1 mm flower buds between male sterile and male fertile plants**

| **Pathway term** | **Rich factor** | **Correct P value** | **Gene number** |
| --- | --- | --- | --- |
| Biosynthesis of unsaturated fatty acids | 0.30303 | 0 | 20 |
| Fatty acid metabolism | 0.142857 | 2.52E-10 | 20 |
| Photosynthesis - antenna proteins | 0.294118 | 0.00079 | 5 |
| Metabolism of xenobiotics by cytochrome P450 | 0.155556 | 0.000807 | 7 |
| Drug metabolism - cytochrome P450 | 0.155556 | 0.000807 | 7 |
| Flavone and flavonol biosynthesis | 0.222222 | 0.010005 | 4 |
| Glutathione metabolism | 0.07 | 0.082559 | 7 |
| Amino sugar and nucleotide sugar metabolism | 0.050314 | 0.271423 | 8 |
| Nitrogen metabolism | 0.107143 | 0.323209 | 3 |
| alpha-Linolenic acid metabolism | 0.065574 | 0.536236 | 4 |
| Cysteine and methionine metabolism | 0.048544 | 0.799165 | 5 |
| Sphingolipid metabolism | 0.090909 | 0.986517 | 2 |
| Glycosphingolipid biosynthesis - ganglio series | 0.2 | 1 | 1 |
| Photosynthesis | 0.050847 | 1 | 3 |
| Phenylalanine, tyrosine and tryptophan biosynthesis | 0.050847 | 1 | 3 |
| Benzoate degradation | 0.166667 | 1 | 1 |
| ErbB signaling pathway | 0.064516 | 1 | 2 |
| Glycosphingolipid biosynthesis - globo series | 0.125 | 1 | 1 |
| Novobiocin biosynthesis | 0.111111 | 1 | 1 |
| Glycosaminoglycan degradation | 0.090909 | 1 | 1 |
