# Supplementary material for: Transcriptomic Analysis of Differentially Expressed Genes during Flower Organ Development in Genetic Male Sterile and Male Fertile Tagetes erecta by Digital Gene-Expression Profiling
Source: PLoS One. 2016 Mar 3;11(3):e0150892. doi: 10.1371/journal.pone.0150892 (PMC4777371; doi:10.1371/journal.pone.0150892)
Supplement: S7 Table — (DOCX) [file pone.0150892.s011.docx]

**S7 Table. The top 16 enriched KEGG pathways of up-regulated DEGs of 1 mm flower buds between male sterile and male fertile plants**

| **Pathway term** | **Rich factor** | **Correct P value** | **Gene number** |
| --- | --- | --- | --- |
| Arginine and proline metabolism | 0.057471 | 0.000262 | 5 |
| Cysteine and methionine metabolism | 0.019417 | 1 | 2 |
| Biosynthesis of amino acids | 0.009772 | 1 | 3 |
| Flavonoid biosynthesis | 0.021739 | 1 | 1 |
| Stilbenoid, diarylheptanoid and gingerol biosynthesis | 0.019608 | 1 | 1 |
| Alanine, aspartate and glutamate metabolism | 0.019608 | 1 | 1 |
| Plant hormone signal transduction | 0.007576 | 1 | 2 |
| Lysosome | 0.016667 | 1 | 1 |
| Glycine, serine and threonine metabolism | 0.013889 | 1 | 1 |
| Glyoxylate and dicarboxylate metabolism | 0.013333 | 1 | 1 |
| Methane metabolism | 0.012821 | 1 | 1 |
| Peroxisome | 0.009434 | 1 | 1 |
| Glycerophospholipid metabolism | 0.007752 | 1 | 1 |
| Phenylpropanoid biosynthesis | 0.007042 | 1 | 1 |
| RNA transport | 0.005319 | 1 | 1 |
| Carbon metabolism | 0.003067 | 1 | 1 |
