# Supplementary material for: Transcriptomic Analysis of Differentially Expressed Genes during Flower Organ Development in Genetic Male Sterile and Male Fertile Tagetes erecta by Digital Gene-Expression Profiling
Source: PLoS One. 2016 Mar 3;11(3):e0150892. doi: 10.1371/journal.pone.0150892 (PMC4777371; doi:10.1371/journal.pone.0150892)
Supplement: S8 Table — (DOCX) [file pone.0150892.s012.docx]

**S8 Table. The top 20 enriched KEGG pathways of down-regulated DEGs of 4 mm flower buds between male sterile and male fertile plants**

| **Pathway term** | **Rich factor** | **Correct P value** | **Gene number** |
| --- | --- | --- | --- |
| Protein processing in endoplasmic reticulum | 0.032129 | 0.246518 | 8 |
| Drug metabolism - cytochrome P450 | 0.066667 | 0.31872 | 3 |
| Metabolism of xenobiotics by cytochrome P450 | 0.066667 | 0.31872 | 3 |
| Flavonoid biosynthesis | 0.065217 | 0.31872 | 3 |
| Plant hormone signal transduction | 0.022727 | 0.939393 | 6 |
| Phenylalanine metabolism | 0.032258 | 1 | 3 |
| Ribosome | 0.018182 | 1 | 6 |
| Proteasome | 0.035088 | 1 | 2 |
| Naphthalene degradation | 0.083333 | 1 | 1 |
| Biosynthesis of unsaturated fatty acids | 0.030303 | 1 | 2 |
| Galactose metabolism | 0.029851 | 1 | 2 |
| Other glycan degradation | 0.071429 | 1 | 1 |
| Degradation of aromatic compounds | 0.066667 | 1 | 1 |
| Phenylpropanoid biosynthesis | 0.021127 | 1 | 3 |
| Glycerolipid metabolism | 0.028169 | 1 | 2 |
| Retinol metabolism | 0.055556 | 1 | 1 |
| Amino sugar and nucleotide sugar metabolism | 0.018868 | 1 | 3 |
| Diterpenoid biosynthesis | 0.047619 | 1 | 1 |
| Chloroalkane and chloroalkene degradation | 0.043478 | 1 | 1 |
| Glutathione metabolism | 0.02 | 1 | 2 |
