# Supplementary material for: Transcriptomic Analysis of Differentially Expressed Genes during Flower Organ Development in Genetic Male Sterile and Male Fertile Tagetes erecta by Digital Gene-Expression Profiling
Source: PLoS One. 2016 Mar 3;11(3):e0150892. doi: 10.1371/journal.pone.0150892 (PMC4777371; doi:10.1371/journal.pone.0150892)
Supplement: S9 Table — (DOCX) [file pone.0150892.s013.docx]

**S9 Table. The top 20 enriched KEGG pathways of up-regulated DEGs s of 4 mm flower buds between male sterile and male fertile plants**

| **Pathway term** | **Rich factor** | **Correct P value** | **Gene number** |
| --- | --- | --- | --- |
| Phenylpropanoid biosynthesis | 0.070423 | 0.004452 | 10 |
| Cysteine and methionine metabolism | 0.058252 | 0.143957 | 6 |
| Fatty acid elongation | 0.088889 | 0.143957 | 4 |
| Stilbenoid, diarylheptanoid and gingerol biosynthesis | 0.078431 | 0.181286 | 4 |
| Phenylalanine metabolism | 0.053763 | 0.235522 | 5 |
| Cutin, suberine and wax biosynthesis | 0.09375 | 0.235522 | 3 |
| Streptomycin biosynthesis | 0.133333 | 0.341326 | 2 |
| Degradation of aromatic compounds | 0.133333 | 0.341326 | 2 |
| Cyanoamino acid metabolism | 0.065217 | 0.39928 | 3 |
| Flavonoid biosynthesis | 0.065217 | 0.39928 | 3 |
| Plant hormone signal transduction | 0.030303 | 0.425011 | 8 |
| Arginine and proline metabolism | 0.045977 | 0.426045 | 4 |
| Nitrogen metabolism | 0.071429 | 0.703909 | 2 |
| Starch and sucrose metabolism | 0.025 | 1 | 6 |
| Butirosin and neomycin biosynthesis | 0.090909 | 1 | 1 |
| Pentose and glucuronate interconversions | 0.031579 | 1 | 3 |
| Naphthalene degradation | 0.083333 | 1 | 1 |
| Retinol metabolism | 0.055556 | 1 | 1 |
| Flavone and flavonol biosynthesis | 0.055556 | 1 | 1 |
| Bisphenol degradation | 0.055556 | 1 | 1 |
